# Integrative multi-omics approach for identifying molecular signatures and pathways and deriving and validating molecular scores for COVID-19 severity and status

**DOI:** 10.1186/s12864-023-09410-5

**Published:** 2023-06-12

**Authors:** Danika Lipman, Sandra E. Safo, Thierry Chekouo

**Affiliations:** 1 Department of Mathematics and Statistics, University of Calgary, Calgary, Canada; 2 Division of Biostatistics, School of Public Health, University of Minnesota, Minnesota, USA

**Keywords:** Integrative analysis, Multi-omics, COVID-19, Pathway analysis

## Abstract

**Background:**

There is still more to learn about the pathobiology of COVID-19. A multi-omic approach offers a holistic view to better understand the mechanisms of COVID-19. We used state-of-the-art statistical learning methods to integrate genomics, metabolomics, proteomics, and lipidomics data obtained from 123 patients experiencing COVID-19 or COVID-19-like symptoms for the purpose of identifying molecular signatures and corresponding pathways associated with the disease.

**Results:**

We constructed and validated molecular scores and evaluated their utility beyond clinical factors known to impact disease status and severity. We identified inflammation- and immune response-related pathways, and other pathways, providing insights into possible consequences of the disease.

**Conclusions:**

The molecular scores we derived were strongly associated with disease status and severity and can be used to identify individuals at a higher risk for developing severe disease. These findings have the potential to provide further, and needed, insights into why certain individuals develop worse outcomes.

**Supplementary Information:**

The online version contains supplementary material available at 10.1186/s12864-023-09410-5.

## Background

COVID-19 has been a concern for medical experts worldwide because of its unpredictable clinical outcomes and varying severity. Deaths from the disease worldwide have been related to acute respiratory distress syndrome (ARDS), a serious lung injury that allows fluids to leak into the lungs [1]. With the outbreak of the disease came a plethora of clinical and molecular data used to understand the mechanisms of the disease. Understanding the biological mechanisms of a disease leads to the development of precise treatments and the prevention or alleviation of severe cases.

Multiple studies have performed analysis of clinical data to analyze severity and long-term effects of the disease [2, 3]. Molecular data have also been analysed, shedding light on the underlying biological mechanisms of the disease and factors contributing to disease severity. Examining the molecular signature of the disease provides information to hypothesize the long-term effects of COVID-19 and develop targeted therapies to alleviate disease severity. Current studies have analysed the molecular signature of the disease through genomics [4], proteomics [5], and multi-omic analyses [6]. A multi-omics study by Overmyer et al., 2020 [7] quantified transcripts, proteins, metabolites, and lipids from patients with COVID-19 and patients experiencing COVID-19-like symptoms. These molecules were associated with clinical outcomes including comorbidity scores, intensive care unit (ICU) status, and disease severity through correlation analysis and machine learning techniques. To our knowledge, there are no papers that take a rigorous integrative multi-omic analysis approach to identify key biomarkers of the disease. Further, molecular scores that combine the effects of multiple biomarkers are yet to be developed for COVID-19 severity. These scores can potentially identify individuals at a higher risk for developing severe COVID-19.

In our previous work [8] we took an alternative approach to analyse the data from Overmyer et al., 2020 [7]. We analysed each view of data separately using stability selection and machine learning techniques to identify molecules of interest for further enrichment analysis, while controlling for a reasonable false detection rate. The main idea behind stability selection, is that rather than performing variable selection on the entire sample, it is performed on multiple subsamples, and variables that are consistently selected are “stable” [9]. Further, we used an unsupervised integrative analysis method to integrate the views of molecular data to analyse simultaneously but were limited in our ability to include clinical covariates, an outcome variable, and prior biological network information.

In this manuscript, our main objective is to use state-of-the-art statistical methods to investigate associations between multi-omics and COVID-19 outcomes (severity and status) and to determine key molecules that drive the relationships between the omics and clinical outcomes. Further, we combined the molecules identified into scores and investigated how well the scores predicted disease severity and discriminated between those with and without COVID-19. We use two rigorous supervised integrative analysis approaches that incorporate clinical covariates and molecular network information to analyze the four views of data: metabolomics, proteomics, lipidomics and RNAseq. When multiple views of data are available for each patient, there is correlation between the views. Analysing each view independently neglects these correlations, while using integrative analysis methods allows to model the dependencies among the views. Modeling correlations or associations between different but related data types could lead to better understanding of disease pathobiology. Integrative analysis methods better model the complexity in multi-omics data compared to separate data analysis. Integrative analysis methods with variable selection are also beneficial in that they are multivariate methods that account for multiple comparisons without using adjusted *p*-values. An integrative analysis method that also incorporates clinical covariates is crucial in understanding how molecular signatures and clinical covariates relate to clinical outcomes. We determined molecules and pathways associated with COVID-19 severity using Bayesian integrative analysis and prediction, BIPnet [10]. The second method we used was sparse integrative discriminant analysis (SIDA) [11]. SIDA combines linear discriminant analysis and canonical correlation analysis to simultaneously model association among views and separation among groups within a view. We determined molecules discriminating between those with and without COVID-19 using SIDA. These methods are able to incorporate pathway information and molecular data with consideration for clinical covariates and outcomes to uncover molecules likely to be key biological markers for COVID-19.

## Results

### Patients characteristics, outcome variables, and omics data

To gain insight into molecular architecture of COVID-19 status and severity, we used publicly available proteomics, metabolomics, RNA sequencing, lipidomics, and clinical data from a study performed by Overmyer et al., 2020. COVID-19 status refers to whether the patient tested positive or negative for the disease, and COVID-19 severity refers to the severity of the disease in the patient measured using HFD45. The study was not long enough to gain insight into long COVID. Our total sample was 123. This comprised 99 patients who tested positive for COVID-19 and 24 patients who experienced COVID-19-like symptoms but tested negative. After filtering the molecular data, our analytical data consisted of 5800 genes, 72 metabolomics features, 264 proteins, and 1015 molecules from lipidomics. Our outcome was either COVID-19 severity or COVID-19 status. A patient’s disease severity was measured by the number of hospital-free days out of 45 days (HFD45). A score of 0 indicates the highest severity with the patient either still being in the hospital after 45 days, or having died before the 45-day period ended. A higher score indicates lower disease severity. A maximum score of 45 would indicate the lowest severity, indicating that out of 45 days, the patient was out of hospital for all 45 days. There is an opposite relationship between the score, and the severity. In the original data collected by Overmyer et al., 2020, there were other scores of severity. However, HFD45 is the most granular, is able to incorporate morbidity, and did not have missing observations so we used this score to analyze disease severity. We do not have a score for severity at the initial time of admission to the hospital, though this would have been more appropriate for analysis to determine the change in severity from time of admission to the hospital. The median HFD45 score was 29. The median age was 63 years, the number of male subjects was 73, sixty-four people were admitted to the ICU, and the median Charlson comorbidity index (CCI) was 3.

### Differential analysis of molecules

**Fig. 1:**
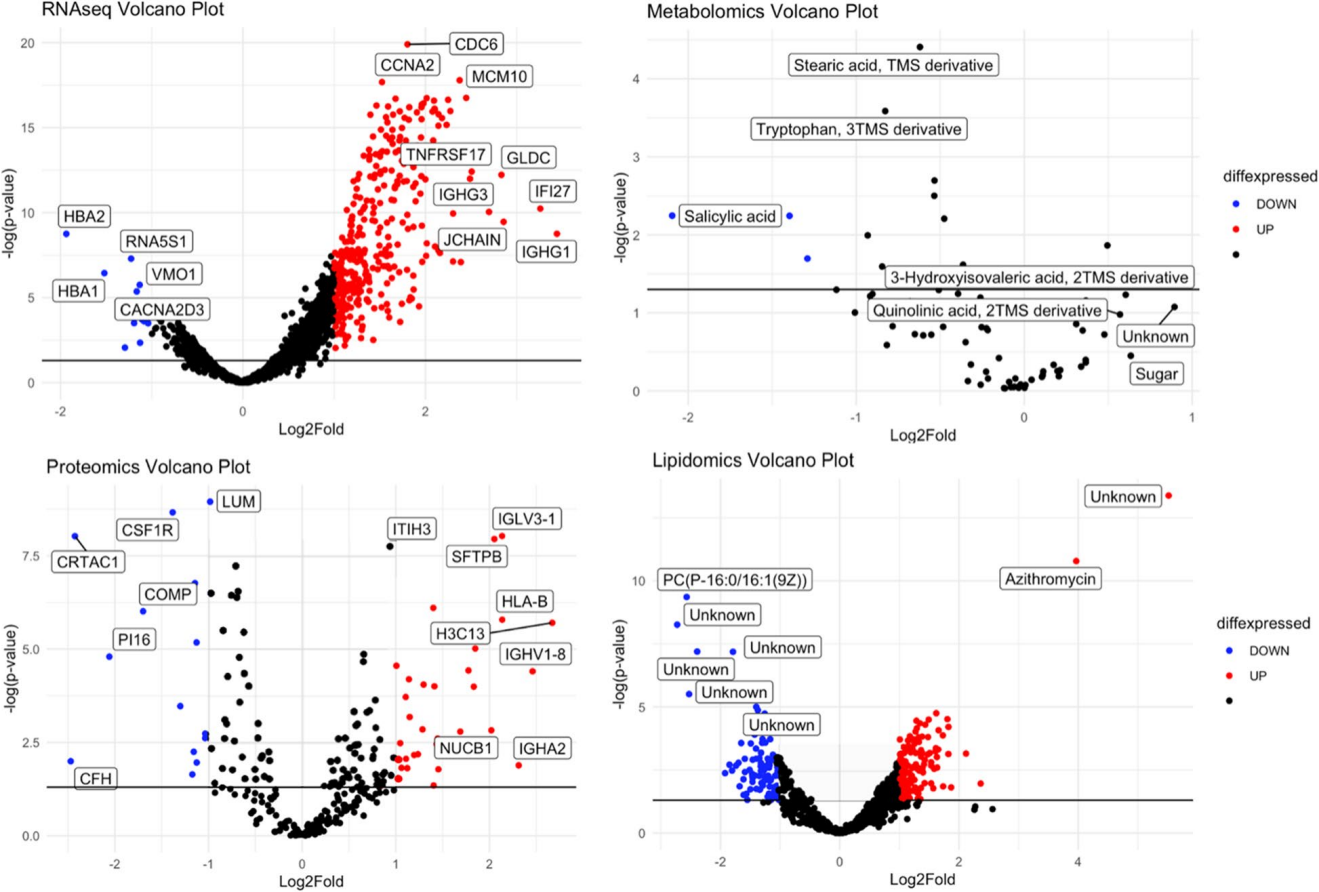
Volcano plot of the filtered RNAseq, metabolomics, proteomics, and lipidomics data. Red represents molecules that are significantly (level 0.05) upregulated in COVID-19, and blue are molecules that are significantly downregulated in COVID-19. Black represents molecules that were not significantly deferentially expressed

**Fig. 2:**
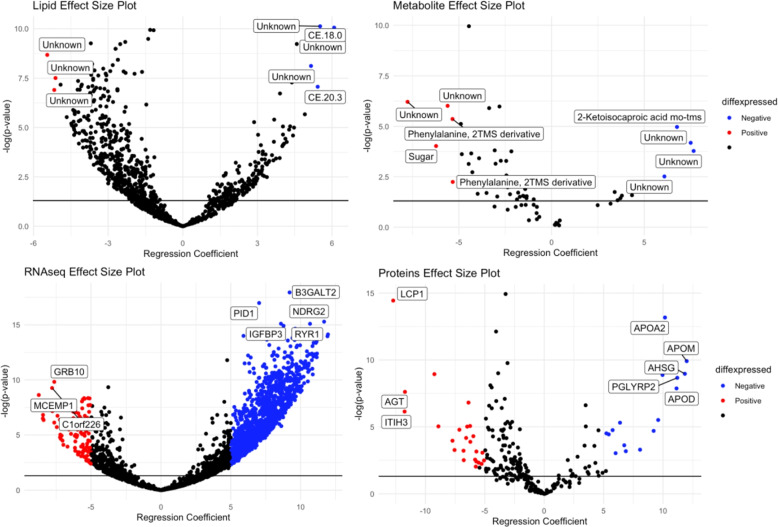
Volcano plot of the filtered RNAseq, metabolomics, proteomics, and lipidomics data. Red represents molecules that are significantly positively associated with disease severity, and blue are molecules that are significantly (level 0.05) negatively associated with disease severity. Black represents molecules that were not significantly differentially expressed

**Fig. 3:**
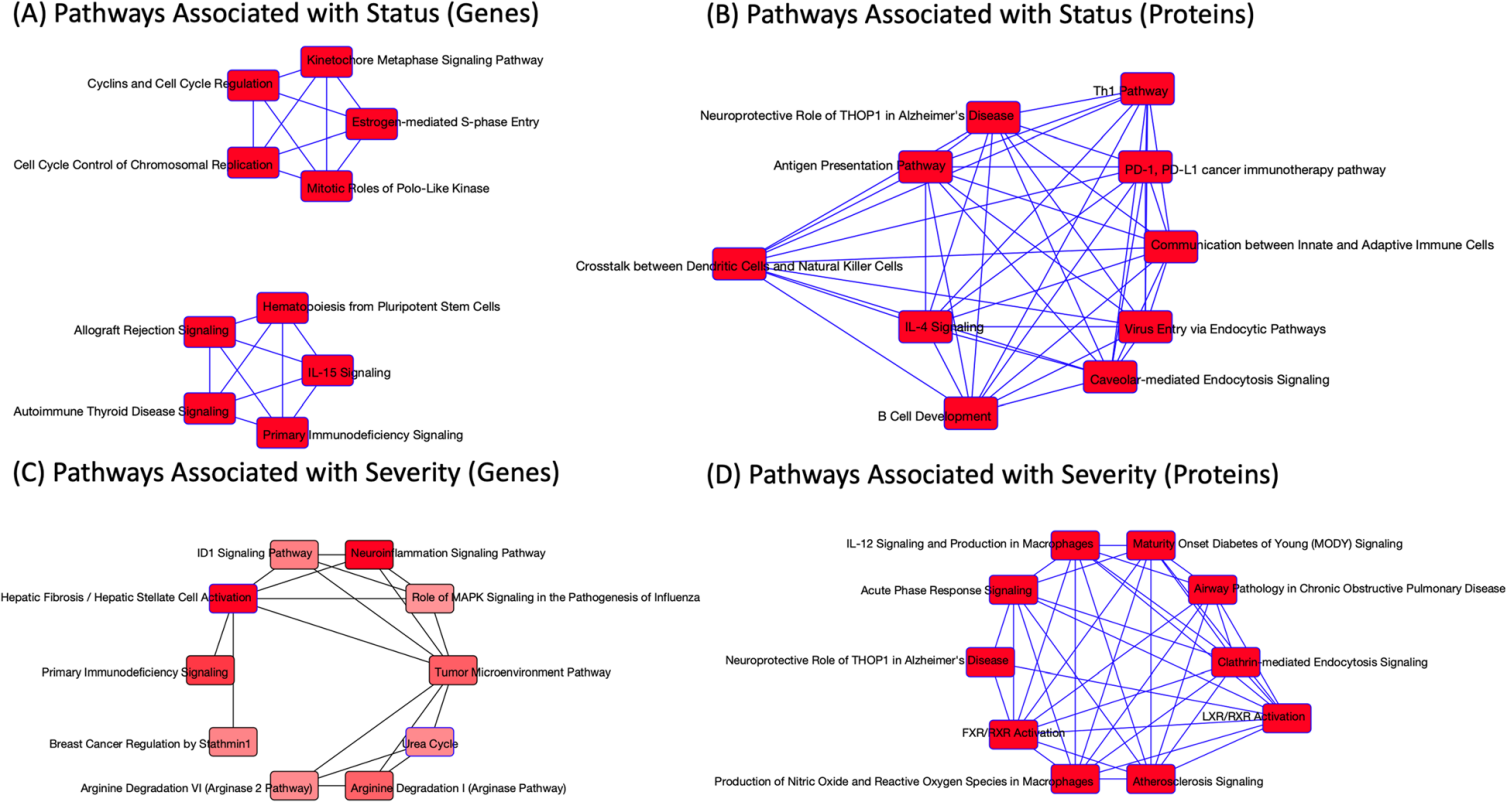
Results of the top 10 pathways associated with COVID-19 status from (**A**) 137 significant genes, (**B**) 16 significant proteins. Results of the top 10 pathways associated with COVID-19 severity from (**C**) 52 significant genes, (**D**) 17 significant proteins

We performed differential analysis (DA) of molecules in each omics data to determine statistically significant molecules associated with HFD45 or disease status. Figure 1 shows volcano plots of up (red) and down (blue) regulated molecules of COVID-19 status. We observed some overlaps (e.g CRTAC1, LUM, CFH, ITIH3, IGLV3-1, and IGHA2) in the proteins determined to be associated with disease status in the DA and in the multivariate integrative analysis, highlighting the potential impact of these molecules in discriminating patients who tested positive from those who tested negative. For insight into the functional classification of these molecules, we performed functional enrichment analysis on the significant molecules (137 genes and 16 proteins, *p*value <0.01) with largest fold changes (absolute value >1.5) using Ingenuity Pathway Analysis (IPA) software. Pathways related to immune function and cell cycle were enriched in our list of genes and proteins [Fig. 3(A-B)]. Figure 2 displays volcano plots of the effect size of each molecule for disease severity. We estimated effect sizes via linear regression– a positive effect size implies that a higher expression level of the molecule is associated with lower severity. We again found some overlaps (LCP1, AGT, ITIH3, APOA2, and APOD) in proteins determined to be associated with severity in the DA and multivariate integrative analysis. Functional enrichment analysis on the statistically significant (p$<0.01$) genes and proteins with largest effect sizes (absolute value $> 7$) (52 genes and 17 proteins) revealed pathways related to immune function and cell cycle. Other pathways related to multiple health conditions such as cancer and immunodeficiency were also enriched. We summarize these results in Fig. 3 (C-D).

### Molecular signatures for COVID-19 severity

#### Molecular panel

In addition to the univariate approach, we considered a multivariate approach to integrating the molecular data, allowing us to investigate the conditional effect of each variable on disease severity given other variables. In particular, a Bayesian integrative analysis method (BIPNet) for simultaneous data integration and outcome prediction (Refer to Methods section), coupled with n-fold (n=61) cross-validation, was used to associate the molecular and clinical data with HFD45, and to determine molecular signatures most likely contributing to the variation in HFD45. The following clinical covariates were included in BIPNet: age, sex, ICU status, and CCI. Of the 5,800 genes, 72 metabolomic features, 264 proteins, and 1,015 lipidomic features used in BIPNet, the number of molecules that were selected in all 61 BIPNet models at a marginal posterior probability (MPP) of at least 0.95 were: 281 genes (MPP $> 0.99$), 21 proteins (MPP $> 0.95$), 8 lipidomics features (MPP$>0.95$), and 3 metabolomics features (MPP $>0.95$). Linear regression models for severity and each selected molecule adjusting for clinical covariates resulted in 3.75 to 12 times more variation in severity explained compared with a baseline clinical model that included age, sex, and CCI (Adjusted R-squared range of 0.15-0.48 versus baseline Adjusted R-squared = 0.04, *p*value<0.05 for all molecules). Of note, 16 genes with the largest effect sizes (>9.5) are all protein-coding genes. Many of the genes we identified are related to immune function, inflammatory response (CCR6, CD4, CD40LG, FCRL3, TLR7), and cell growth (DYRK2, MSX2). Further, IPA found that many proteins identified are related to the insulin regulation biological process (CFD, IGFBP2, FETUB), as was also noted in COVID-19 research paper [12], and inflammation and immune response (S100A8, SAA2, DEFA1, LYZ, B2M, FETUB, LCN2).

#### Molecular scores

We further constructed molecular scores based on a panel of molecules or pathways from Ingenuity Pathway Analysis of candidate molecules. We investigated whether these molecular scores, in addition to clinical factors, are able to predict severity better than clinical factors alone.

##### Scores based on pathways

**Fig. 4:**
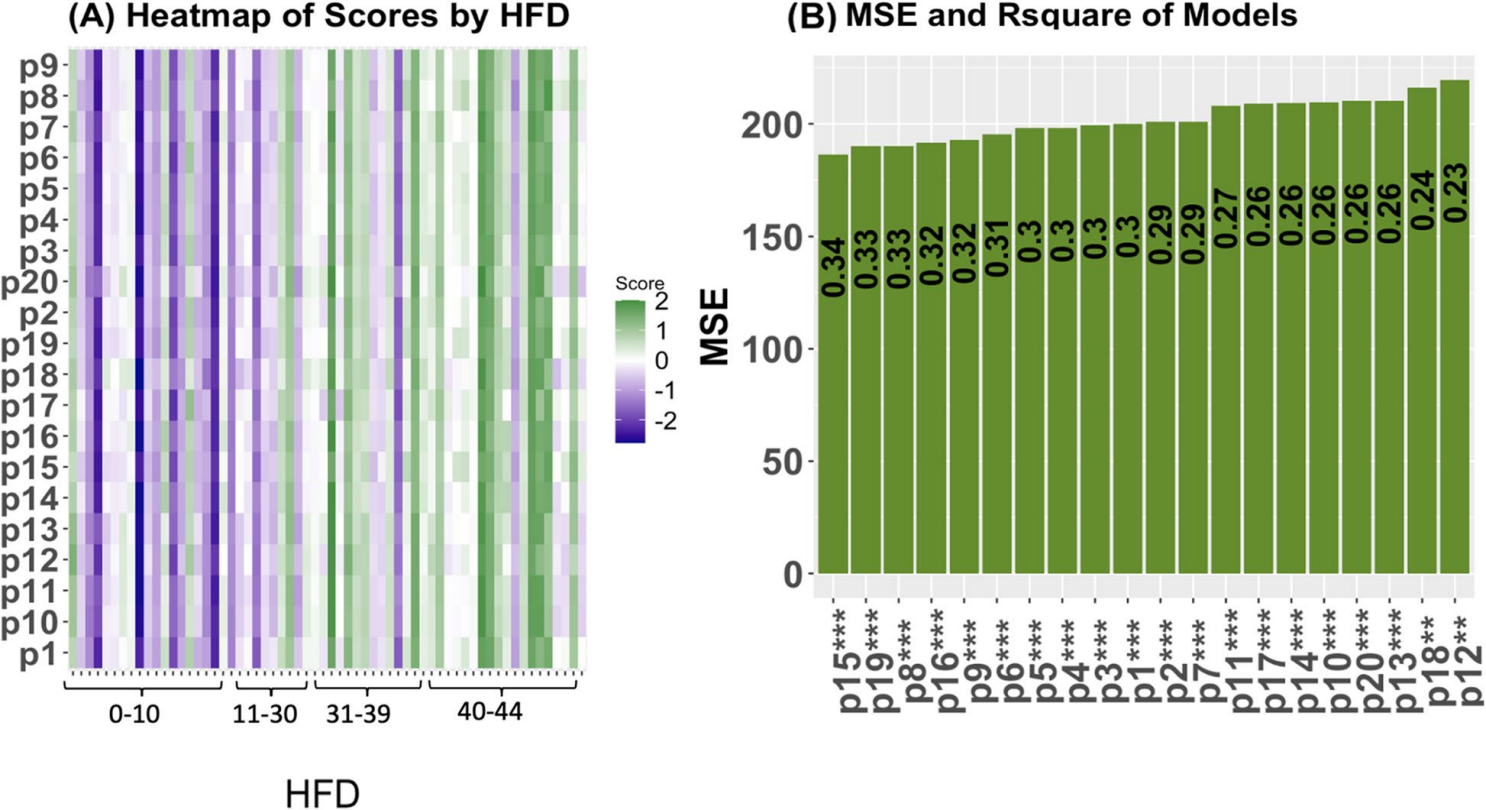
Results from the BIPnet gene pathway scores. (**A**) Heatmap of the scores across HFD (**B**) Plot of MSE of the models with and without adjusting for clinical covariates. Labeled with the adjusted R squared from the regressions. *P*values denoted as: * $\le 0.05$, ** $\le 0.01$, ***$\le 0.0001$. Pathway names are as follows: p1=Th1 Pathway, p2= Th1 and Th2 Activation Pathway, p3=Th2 Pathway, p4= ICOS-ICOSL Signaling in T Helper Cells, p5=T Cell Receptor Signaling, p6=PD-1, PD-L1 cancer immunotherapy pathway, p7=FAK Signaling, p8=Primary Immunodeficiency Signaling, p9=CD28 Signaling in T Helper Cells, p10=G-Protein Coupled Receptor Signaling, p11=CREB Signaling in Neurons, p12=CREB Signaling in Neurons, p13=Phagosome Formation, p14=Non-Small Cell Lung Cancer Signaling, p15=Calcium-induced T Lymphocyte Apoptosis, p16=Role of NFAT in Regulation of the Immune Response, p17=Natural Killer Cell Signaling , p18=Protein Kinase A Signaling, p19=PKC$\theta$ Signaling in T Lymphocytes, p20=Breast Cancer Regulation by Stathmin1

We performed Ingenuity Pathway Analysis (IPA) on the selected proteins and genes to provide further insight into the driving networks. Pathways are groups of molecules that work toward a certain process [13]. We used all 281 selected genes for pathway analysis. Results from IPA on the 281 genes are summarised in Supplementary Table 1. Although we used IPA to access network information, there are other easily accessible methods of gaining network information of molecules, for example KEGG [14]. We performed enrichment analysis in IPA, though there are other methods for enrichment analysis such as the software GSEA (https://www.gsea-msigdb.org/gsea/index.jsp). These results provide insight into possible long-term consequences of severe cases, as well as biological functions being affected. We constructed scores for each pathway using effect sizes for each molecule in the pathway as determined in the univariate regression models (Supplementary Figs. 1-4). Details on how scores are created are provided in Conclusions section. Supplementary Table 2 contains the adjusted R-square value from the regressions using pathway scores (see methods), and the MSE of the models. Figure 4 shows (A) heatmaps of the relationship between the pathway scores and HFD45 and (B) a histogram of the MSE labeled with adjusted R-squared. The top networks based on MSE and adjusted R squared included the primary immunodeficiency signaling pathway, CD28 signaling in T helper cells, calcium-induced T lymphocyte apoptosis, the role of NFAT in the regulation of immune response, and PKC signaling in T lymphocytes. These pathways explained 8 to 8.5 times the variation in severity compared with the baseline clinical model. These findings suggest that pathways regarding T cells and immune response are strongly associated with disease severity, which is to be expected. We discuss these pathways further in the Discussion section.

**Fig. 5:**
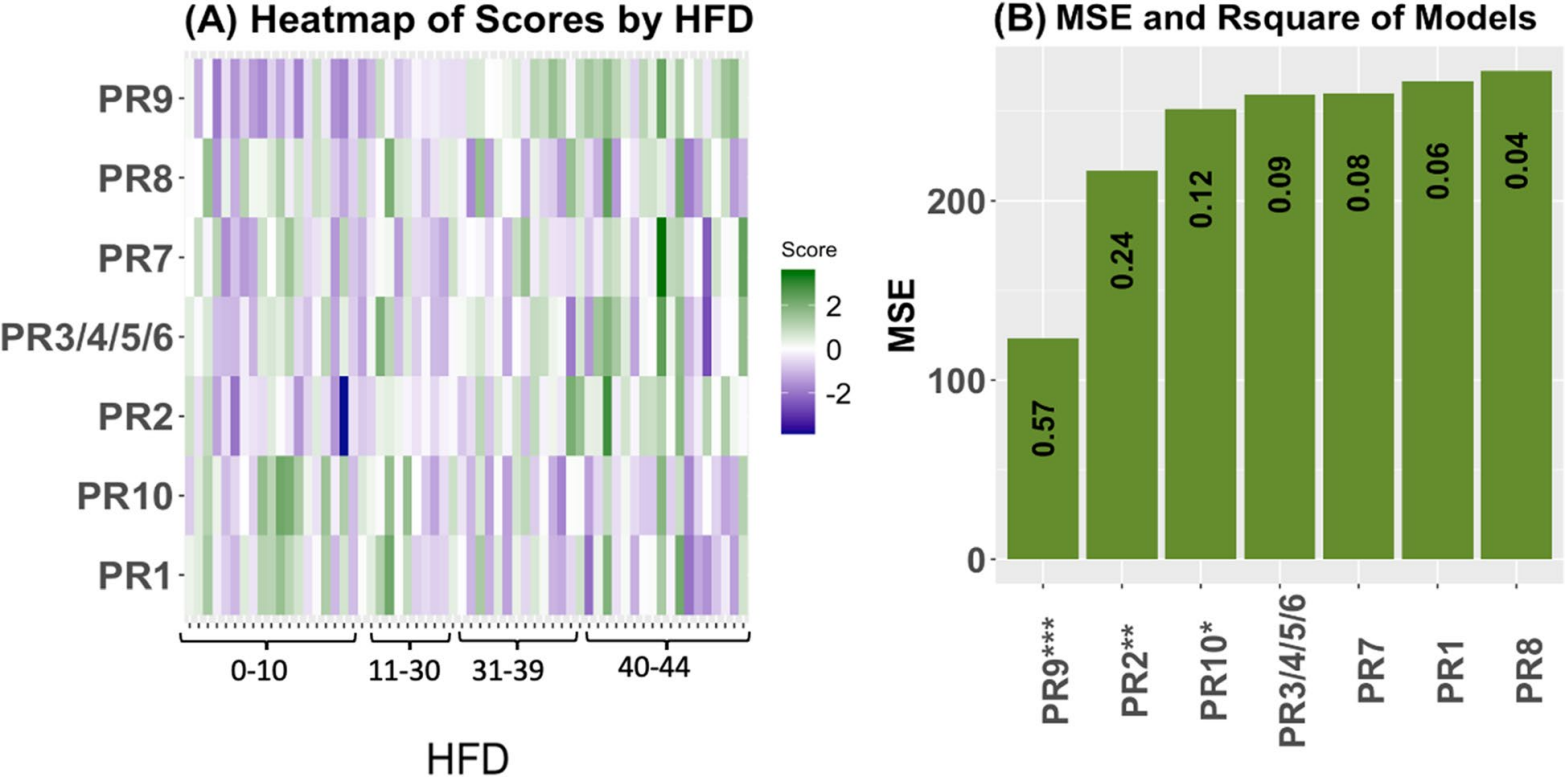
Results from the BIPnet protein pathway scores. (**A**) Heatmap of the scores across HFD (**B**) Plot of MSE of the models with and without adjusting for clinical covariates. Labeled with the adjusted R squared from the regressions. *P*values denoted as: * $\le 0.05$, ** $\le 0.01$, ***$\le 0.0001$. Pathway names are as follows: PR1=LXR/RXR Activation, PR2=FXR/RXR Activation, PR3=Atherosclerosis Signaling, PR4=IL-12 Signaling and Production in Macrophages, PR5=Production of Nitric Oxide and Reactive Oxygen Species in Macrophages, PR6=Clathrin-mediated Endocytosis Signaling, PR7=Airway Pathology in Chronic Obstructive Pulmonary Disease, PR8=Acute Phase Response Signaling, PR9=Maturity Onset Diabetes of Young (MODY) Signaling, PR10=Neuroprotective Role of THOP1 in Alzheimer’s Disease

We repeated the same process for the proteins selected in BIPnet. The results from IPA on the 21 proteins are summarised in Supplementary Table 3, where we observe the top 10 significant canonical pathways. We developed a score for each network that had unique molecules selected. Refer to Supplementary Table 4 for results. A visual of the scores’ associations with disease severity, as well as the MSE and adjusted R-squared is presented in Fig. 5. The MODY (maturity-onset diabetes of young) pathway had the most obvious association with disease severity and lowest MSE and highest adjusted R squared (explaining 14.25 times the variation in the HFD45 than the baseline clinical model). From the models, the most significant pathways are MODY signaling, the neuroprotective role of THOP1 in Alzheimer’s disease, and FXR/RXR Activation pathways. We discuss these results further in the Discussion section.

##### Scores Based on a Panel of Molecules

**Fig. 6:**
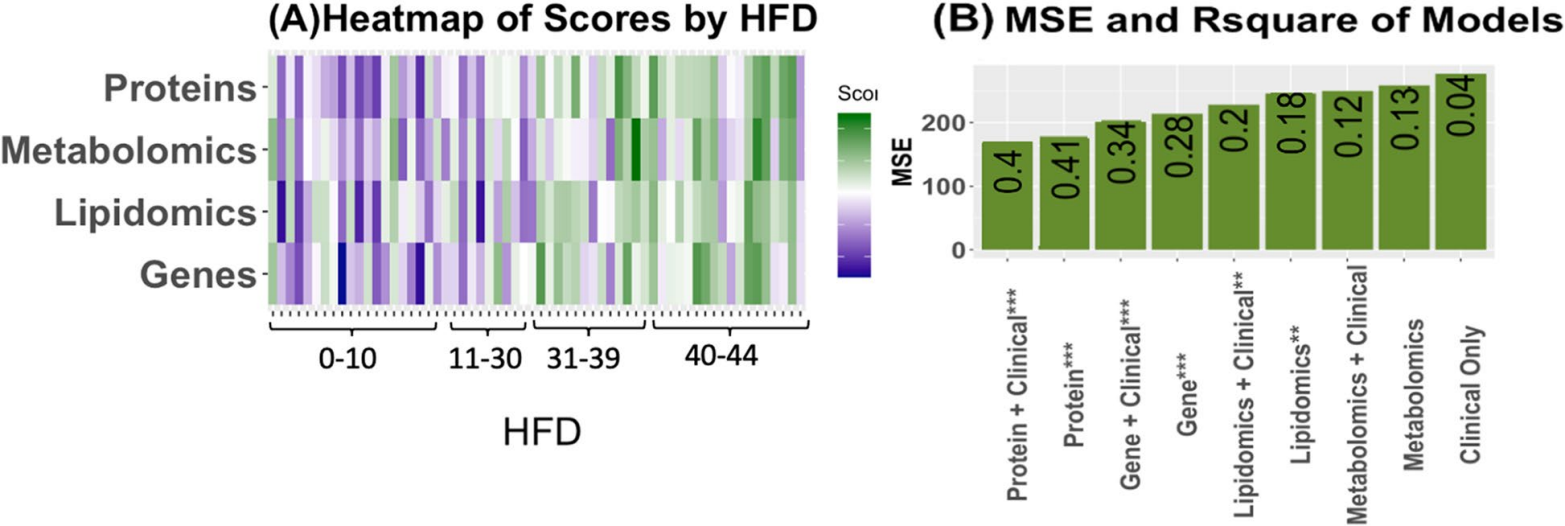
Results from the BIPnet scores. (**A**) Heatmap of the scores across HFD, a pattern is most noticeable in the protein and lipid scores. (**B**) Plot of MSE of the models with and without adjusting for clinical covariates. Bars are ordered in ascending order of MSE. Labeled with the adjusted R squared from the regressions. *P*values denoted as: * $\le 0.05$, ** $\le 0.01$, ***$\le 0.0001$

We used the effect sizes (Supplementary Figs. 1-4) of the molecules to construct scores for each view of data. A score comprised of all genes performed poorly and had convoluted utility. To improve predictive performance and hone in on key molecules the genes with the largest effect sizes were used (16 genes with weights>9.5. Cutoff selected based on graphical analysis choosing approximately the top 5$\%$). We present the molecules used to create the scores in Supplementary Table 5. The scores demonstrate their predictive value in disease severity and identify patients at high risk of developing severe cases. We fit separate models with/without adjusting for clinical covariates for each datatype score and present the results of the coefficient of the score, its *p*value, and 95 percent confidence interval in Supplementary Table 6. We provide a model fit using clinical covariates (age, sex, and CCI) for comparison of MSE and adjusted R-squared. The scores with the best accuracy are the protein scores (explaining 10 times the variation than the clinical model) and gene scores(explaining 8.5 times the variation than the clinical model). We provide a visual of the relationship between HFD and the scores and a comparison of the models’ adjusted R squared and MSE in Fig. 6. These scores generally provide adjusted R-squared values which are higher, and MSEs which are lower than the models based on individual molecules, with few exceptions. It is evident the scores we have created are highly predictive of disease severity and can identify high-risk individuals. We observed that the association of proteins and HFD45 was the strongest, the adjusted R-square largest, and the mean squared error the smallest (Fig. 6). From the heatmap, a lower protein score was associated with more severe disease. From Supplementary Fig. 4, protein S100A8 had a large negative coefficient (based on the weights), which indicated that holding all other proteins in the score constant, an elevation in this protein decreases the score and hence increases severity. Interpretations for the other molecules in the scores are similar. In Supplementary Table 7, we include a summary of the model using all scores as predictors. This model had an R squared value of 0.44, and an MSE of 151.83 and was the best model, explaining 11 times the variation in severity than the baseline clinical model. From the model containing all scores, the proteins are still the most significant.

### Molecular signatures for COVID-19 status

#### Molecular panel

We identified multi-omics signatures associated with COVID-19 status by applying n-fold cross-validation (n = 61) with SIDA on the training set. We investigated the effects of each molecule on disease status conditional on all other molecules of each view. The cross-validated misclassification rate of the SIDA models was 0.07, and we consistently selected 44 proteins, 20 genes, 7 metabolites, and 3 lipids in all 61 models. We performed logistic regression with each molecule as a predictor in its own model adjusting for clinical covariates. The AUCs from these models are as high as 1.5 times the AUC of the baseline clinical model. We present the effect sizes and significance levels from these models in Supplementary Figs. 5-8. We identified recurring functions of selected proteins as neural functions and conditions (CHL1, ITI3) and immune response (LCP1, IGLV3-1). As for selected genes we observed molecules related to immune function (ILDR1). One particularly interesting gene we selected in SIDA is OR52K1, which is related to the perception of a smell. This is interesting as patients with COVID-19 commonly experience a loss of smell.

#### Molecular scores

As with the disease severity section, we constructed molecular scores based on a panel of molecules or pathways from Ingenuity Pathway Analysis of candidate molecules. We investigated whether these molecular scores, in addition to clinical factors, are able to predict disease status better than clinical factors alone.

##### Scores based on pathways

**Fig. 7:**
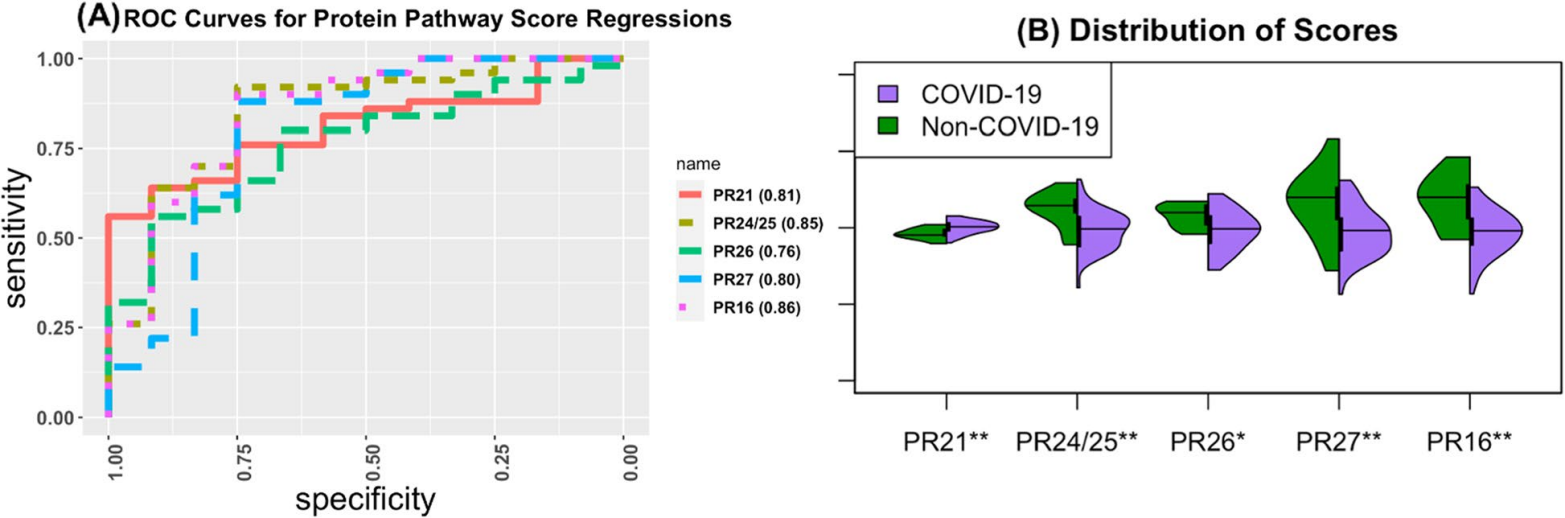
Results from the SIDA protein pathway scores. (**A**) The ROC curves for each of the score regression models which was significant at a level of 0.05. AUC is presented in the legend. (**B**) The distribution of the scores across the COVID and non-COVID individuals in the testing dataset. *P*values denoted as: * $\le 0.05$, ** $\le 0.01$, ***$\le 0.0001$. PR16 = Maturity Onset Diabetes of Young (MODY) Signaling, PR21 = Complement System, PR24 = Atherosclerosis Signaling , PR25 = IL-12 Signaling and Production in Macrophages, PR26 = Aldosterone Signaling in Epithelial Cells, PR27 = PPARa/RXRa Activation

We performed IPA on the 44 proteins and 20 genes selected in SIDA. Supplementary Table 8 contains the top canonical pathway for genes. Only one gene pathway (tumor microenvironment pathway) is significant at a level of 0.05 from Fisher’s test. Supplementary Table 9 contains results from the regression using the score created from molecules in this pathway adjusting for clinical covariates. We determined this pathway score does not have a strong association with COVID-19 status. We provide results from IPA for the 44 proteins in Supplementary Table 10 indicating 17 pathways are significant at a level of 0.05. We present the results from the regressions using the scores from the pathways in Supplementary Table 11. Figure 7 contains the ROC curves for each of the pathway scores, as well as the distribution of the scores across the COVID and non-COVID individuals in the testing dataset. From our score regressions, the atherosclerosis signaling pathway, IL-12 signaling and production in macrophages, PPARa/RXRa activation, MODY signaling, and complement system had the strongest associations with AUCs being 1.36-1.45 times the AUC of the clinical baseline model. Distribution plots of the molecules in these pathways by COVID-19 status are available in Supplementary Figs. 9-11 where we observed the molecules in the selected pathways had different abundances depending on COVID-19 status. From our analysis using SIDA and BIPnet there was some overlap in the molecules we selected. Specifically, we selected the proteins CRTAC1, LUM, APOA2 in relation to both disease status and severity. We also identified pathways that are enriched in both severity and status. From the proteins we selected in SIDA and BIPnet the pathways LXR/RXR activation, FXR/RXR activation, atherosclerosis signaling, IL-12 signaling and production in macrophages, acute phase response signaling, maturity-onset diabetes of young (MODY) signaling, and neuroprotective role of THOP1 in Alzheimer’s disease were selected. We discuss these pathways further in the Discussion section.

##### Scores Based on a Panel of Molecules

**Fig. 8:**
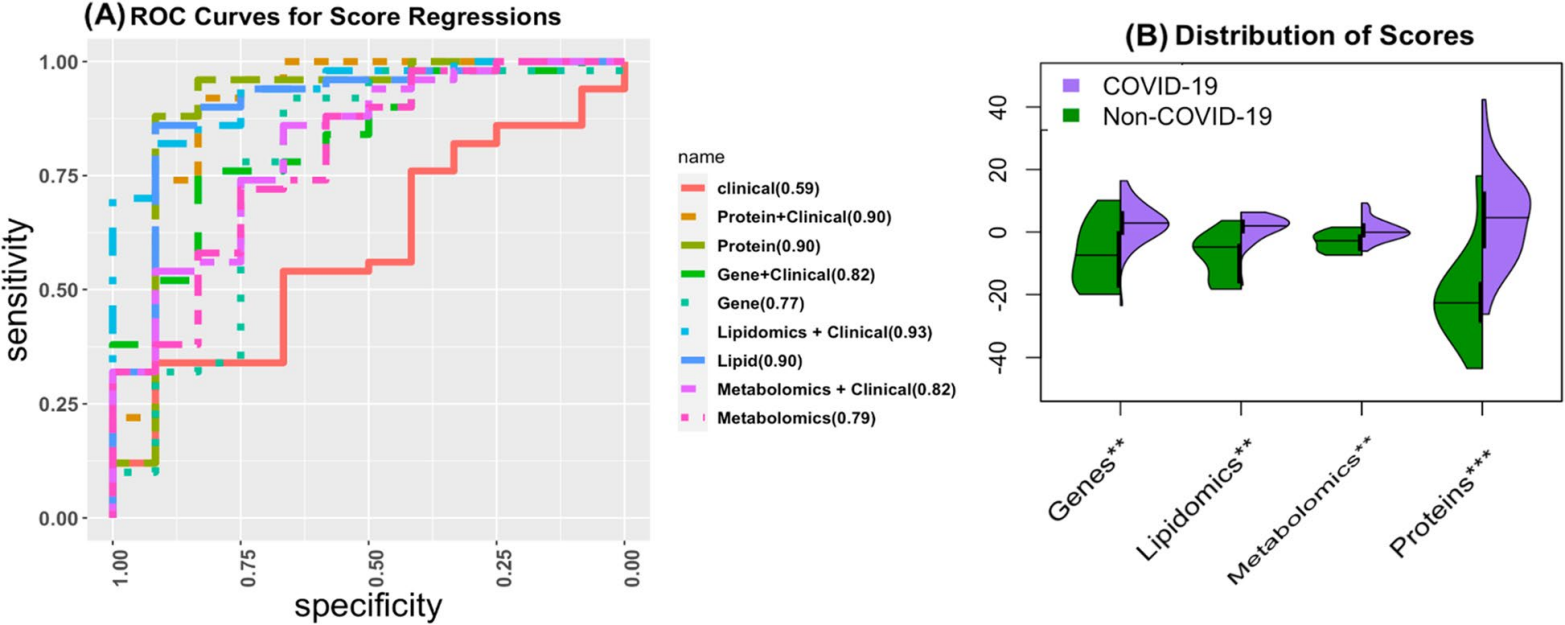
Results from the SIDA scores. (**A**) The ROC curves for each of the score regression models with and without adjusting for clinical covariates. AUC is indicated in the legend. (**B**) The distribution of the scores across the COVID and non-COVID individuals in the testing dataset. *P*values denoted as: * $\le 0.05$, ** $\le 0.01$, ***$\le 0.0001$

We used the effect sizes from the logistic regression models to create a score composed of a linear combination of selected molecules for each view of data. Initially, a score using all genes and proteins was created but had a poor predictive performance. For improved prediction and utility, the number of genes and proteins used in the scores was reduced to 10 for genes, and 11 for proteins as these molecules had the highest significance (*p*value <0.01). Supplementary Table 12 has the list of molecules used in creating the score. In addition, we have provided the distribution of these molecules for each dataset by COVID-19 status in Supplementary Figs. 12-15. We fit regression models using these scores with/without adjusting for clinical covariates and present the AUC, coefficients of scores, and *p*values for the scores in Supplementary Table 13. Figure 8 contains the ROC curves and AUC for each of the scores with and without adjusting for clinical covariates, as well as the distribution of the scores across the COVID and non-COVID individuals in the testing dataset. We included the model with only clinical covariates (age, sex, CCI, and ICU status) for comparison. It is evident that the scores we have created are significantly associated with COVID-19 status with the AUCs of the model ranging from 1.3 to 1.57 times the AUC of the clinical baseline model. The most accurate model provides discriminatory accuracy higher than any individual molecule. In particular, the protein score we developed has the best ROC curve and the best separation of classes. This means we have identified a significant panel of molecules which can accurately classify COVID-19 patients.

### Validation of results

We used three independent datasets to validate the scores we created in Molecular scores section. All datasets used are open source and had the molecules log2-transformed and standardized to have mean zero and variance one.

#### Validation of proteomics severity scores

**Fig. 9:**
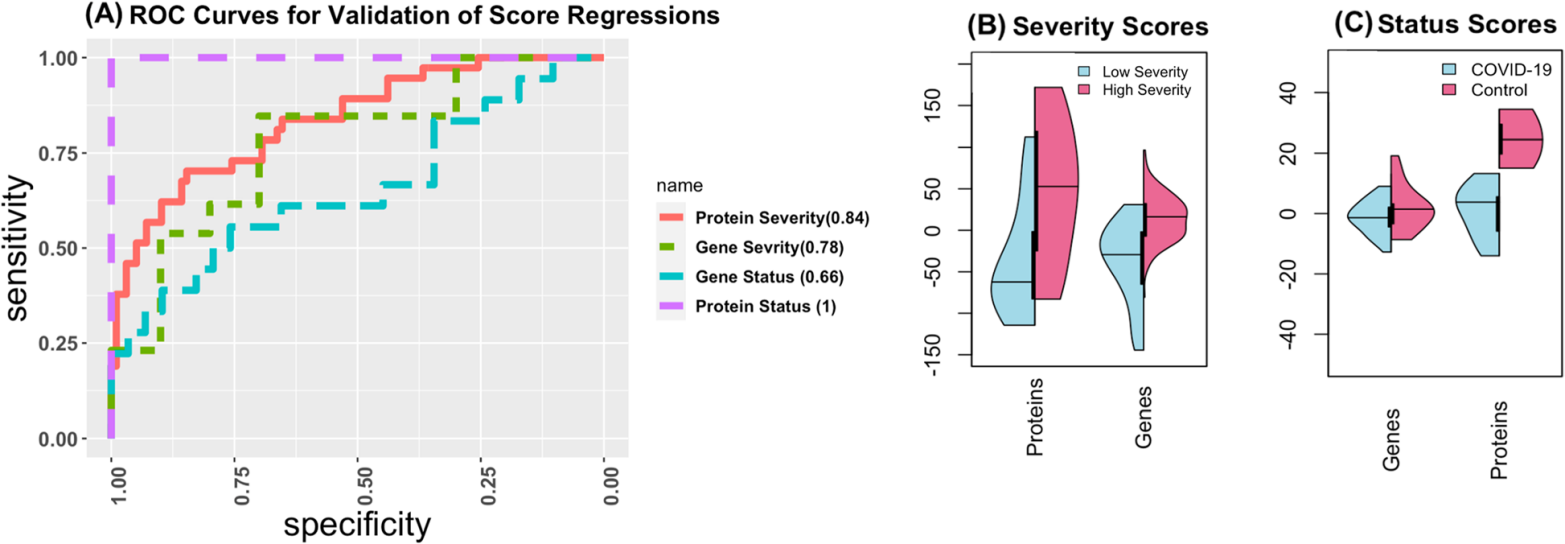
Results from validation. (**A**) The ROC curves for each of the score validation regression models. AUC is indicated in the legend. (**B**) The distribution of the scores by Severity group, (**C**) The distribution of the scores across the COVID and non-COVID individuals

To validate proteomics scores for COVID-19 severity, we used the data from (Wu et. al) [6] and weights estimated in Molecular scores section to obtain a score for each of the 135 samples aged between 19 and 70. The samples in this dataset did not have severe comorbidities. The severity outcome in this dataset is measured as an ordinal response: asymptomatic (n=60), mild (n=38), severe (n=22) , and critical (n=15), which we grouped into two classes: asymptomatic-mild, and severe-critical, to be consistent with our outcome. Since the proteomics data did not contain all of the proteins we used to develop our score, we could only validate the score comprised of the following proteins: AGT, APOA2, APOD, B2M, CFD, CST3, DEFA1, FETUB, IGFBP2, LCN2, LUM, LYZ, PTGDS, RNASE1, S100A8, SAA2, TNC (4 missings). Therefore, we computed the scores for the validation dataset using only the weights we found for these proteins. We performed logistic regression with the new scores as a predictor. The AUC resulting from the logistic regression was 0.837 demonstrating that the derived score is strongly associated with disease severity even in an independent dataset. The ROC plot from this dataset and the distribution of the scores across the classes are available in Fig. 9.

#### Validation of genomics severity scores

To assess the validity of our genomics score for severity, we used data obtained from (Wargodsky et. al) [15]. The data consist of 13 critically ill patients, and 10 patients with mild to moderate outcomes over the age of 18 who tested positive for COVID-19. Due to missing data for some genes, we could only validate the score derived in Molecular scores section using the following molecules: CCR6, CD4, CD40LG, CX3CR1, DYRK2, FCRL3, MAN1C1, P2RY10, TLR7, ZNF549, ZXDB (missing 5). Using logistic regression with the score as the predictor, the AUC was 0.7769, demonstrating that the score is able to predict severity fairly well when applied to this independent dataset.

#### Validation of gene scores for COVID-19 status

We used data from (Blanco-Melo D et. al) [16] to validate our gene score for disease status. The subset of data we used consisted of 18 samples infected with COVID-19 and 29 samples that were mock treated, all coming from four different cell lines. Due to missing data, we could only use the weights of the following genes to create a new score for each sample: CILP2, HLA-G, LCNL1, MMP17, VMO1, and ILDR1 (4 missing). A logistic regression with only the cell line as predictor produced an AUC of 0.5977, while AUC with the cell line and gene score produced had an AUC of 0.6571. The AUC with only the score is 0.6034. This demonstrates that the score is able to improve the discrimination between COVID-19 and non-COVID-19 status. We believe the accuracy would be further improved if the missing genes could have been included in our validation.

## Discussion

The objective of this manuscript is to use state-of-the-art statistical methods to investigate associations between multi-omics and COVID-19 outcomes. From our analysis we have determined a panel of molecules and molecular pathways with strong associations to clinical outcomes. These molecules and pathways can be used to assess patient risk and develop targeted treatment plans. We discuss three main findings we have from the analysis. The first point we will discuss is the pathways regarding immune function which we found to be significant from IPA on molecules selected from BIPnet and SIDA. The second main finding we discuss is pathways that are not directly related to immune function but were also found to be related to COVID-19 status and severity. The final discussion point pertains to the panel of molecules which we determined to discriminate between those with and without COVID-19, and have strong associations with disease severity.

### Enriched pathways related to immune function

We determined from the BIPnet analysis that many pathways surrounding T cells are related to COVID-19 severity. In particular, the ICOS-ICOSL signaling in T helper cells, PKC signaling in T lymphocytes, T cell receptor signaling, calcium-induced T lymphocyte apoptosis, and CD28 signaling in T helper cells were all determined to be related to COVID-19 severity. In relation to current literature, these findings are noteworthy. In particular, one paper [17] specifically investigated the T-cell immune response against COVID-19, and found that T-cell responses were impaired in severe COVID-19 cases, suggesting a possible therapeutic mechanism to reduce COVID-19 severity.

From the analysis we observed the PD-1, PD-L1 cancer immunotherapy pathway is associated with COVID-19 severity. Research has been conducted on the possible relationship between PD-L1 and COVID-19. PD-1 (programmed cell death protein 1) and PD-L1 (programmed cell death ligand 1) play a role in immune response and it has been found that PD-L1 dysregulation is associated with COVID-19 [18]. In particular, higher levels of PD-L1 are associated with more severe cases and even death from COVID-19. Sabbatino F. et al. [18] found associations with age and lymphocyte levels of the patients. This pathway is also responsible for T cell activation, providing further evidence about the association of T cell functions/processes and COVID-19 severity.

We determined two pathways related to macrophage processes were enriched: IL-12 signaling and production in macrophages and the production of nitric oxide and reactive oxygen species in macrophages. In addition, we found the acute phase response signaling pathway significant with proteins selected in SIDA and BIPnet. We found this pathway to be enriched in our previous work [8].

### Enriched pathways unrelated to immune function

The first pathways we discuss which are not directly related to immune function are the LXR/RXR activation, FXR/RXR activation, atherosclerosis signaling, maturity-onset diabetes of young (MODY), and neuroprotective role of THOP1 in Alzheimer’s disease pathways. We determined these pathways are associated with COVID-19 in our previous work [8] and were also found to be enriched from IPA on proteins we selected from both SIDA and BIPnet. The atherosclerosis signaling pathway is of particular interest, as many studies have investigated the link between COVID-19 with venous and arterial circulations. One paper, in particular, examined the relationship between COVID-19 and atherosclerosis, observing the similarities and differences in mechanisms of the diseases [19]. Maturity-onset diabetes of the young (MODY) is a form of diabetes mellitus usually diagnosed in young adulthood [20]. This pathway is interesting as there are cases to show that diabetes may be a risk factor for more severe cases of COVID-19, but may also be a consequence of COVID-19 infection [21]. To our knowledge, there has not been a thorough analysis of the relationship between MODY and COVID-19 but there has been some evidence to show that COVID-19 affects glucose metabolism, which is related to diabetes.

The relationship between COVID-19 and glucose metabolism is evident in our analysis. IPA on proteins selected from SIDA revealed that the gluconeogenesis I and glycolysis I pathways are determined to be associated with COVID-19. As mentioned, research suggests that there is a relationship between COVID-19 and blood glucose metabolism [22]. Current studies also investigated the effect of glucose metabolism on T cell function in COVID-19, and it has been suggested that targeting glucose metabolism may be a viable treatment to reduce the severity of COVID-19 [23]. Other pathways we determined to be associated with COVID-19 that relate to glucose metabolism are the LXR/RXR activation pathway, and the FXR/RXR activation pathway. Another interesting aspect of the FXR/RXR activation pathway is its association with liver disease [24]. Current research suggests that individuals with liver disease are more at risk of severe COVID-19 [25]. This is of interest as there has been what appears to be an increased risk of liver damage in patients who have COVID-19, and this pathway could be related to this outcome [26]. To our knowledge, the relationship between this pathway and liver damage from COVID-19 has not been extensively researched.

A surprising pathway we found associated with COVID-19 severity is the neuroprotective role of THOP1 in Alzheimer’s disease, however, recent studies have shown that Alzheimer’s-like signaling is occurring in the brains of COVID-19 patients [27]. It has been suggested that the brain fog patients experience after COVID-19 may be a form of Alzheimer’s, although more research into this is required to come to any definitive conclusions.

### Novel panel of molecules associated with COVID-19

The genes we determined to be most strongly associated with COVID-19 severity from BIPnet are available in Supplementary Table 1. The score we created from these molecules is strongly associated with COVID-19 severity, ensuring us that BIPnet is able to accurately select predictors for our response. Of these genes, CCR6, CD4, CD40LG, CX3CR1, DYRK2, FCRL3, MAN1C1, P2RY10 and TLR7 have been identified as associated with COVID-19 in other research [28–35], however the other genes appear to be novel. Further investigation into these genes may provide insight into the mechanisms of COVID-19. The proteins we determined to be most strongly associated with COVID-19 severity by BIPnet are available in Supplementary Table 1. CRTAC1, APOD, CFD, AGT, S100A8, RNASE1, SAA2, MRC1, TNC, DEFA1 , LYZ, B2M, DAG1, FETUB, APOA2, and LCN2 have all been identified as associated with COVID-19 in other research [36–44], but the remaining proteins appear to be novel to our research. The score we created from these proteins was found to have a high predictive power of COVID-19 severity. These findings suggest that the molecules we have identified and used to develop a score could be used to accurately identify high-risk individuals. Note that the lipids and metabolites selected via BIPnet are unidentified molecules so unfortunately cannot provide us with insight into the disease. In addition to the molecules we selected from BIPnet, we also have a panel of molecules identified by SIDA. The most significant proteins we found by SIDA are provided in Supplementary Table 5. From these proteins CRTAC1, IGLV3-1, HRG, HSPB1, LCP1, ITIH3, and APOA2 have all been identified as correlating with COVID-19 in other research, but to our knowledge, the rest are novel [45–48]. One new molecule we identified that may be of particular interest is the LUM protein, which as selected in SIDA and BIPnet. This protein is related to the cornea, and metabolism, and further investigation into this protein’s association with COVID-19 could be interesting. Referring to Supplementary Fig. 11, it is apparent there is a large difference in the expression of this protein between the COVID-19 and non-COVID-19 groups. The score that we created from these proteins substantially increased AUC in the model, which indicates they are particularly predictive of COVID-19. The genes we selected from SIDA and used to create a score are provided in Supplementary Table 5. Of these genes, there is research to identify HLA-G as being associated with COVID-19, however, the rest of these molecules appear to be novel [49]. One molecule that is of interest here is the MMP17 gene, as this is a matrix metalloproteinase gene, and there is evidence to show that other matrix metalloproteinase molecules are related to neurological complications resulting from COVID-19 [50]. Further research into the novel molecules discovered in this analysis can help us understand the underlying signature of the disease and identify treatments to reduce the severity. We were able to validate some of our results by calculating the protein and gene scores for severity and status on independent datasets. Not all of the molecules in our scores could be validated due to missing data in the independent datasets.

Some limitations of the study are that we have unidentified lipids and metabolites which are determined to be significant. It would be of interest to identify these molecules as they may provide more insight into COVID-19. In addition, we have excluded many clinical covariates due to missing data, however, the use of multiple views of molecular data allows us to still make accurate analyses and conclusions. We considered comorbidities of patients in the analysis by incorporating CCI as a clinical covariate, though for future studies more detailed information about the comorbidities patients are experiencing (for example, if the patient has heart or respiratory conditions) would allow more accurate results in determining what is strictly associated with COVID-19. In addition, we are missing information regarding patient treatment. However, as mentioned in the original study, Azithromycin was used as treatment in many patients before enrolment in the study, and hence was found to be falsely associated with COVID-19. As such, this molecule was excluded from the analysis, as it is not actually associated with COVID-19. Future studies that incorporate treatment information may allow more comprehensive analysis of treatment effects on severe disease. Blood samples were taken at time of admission to the hospital, so we do not expect to see a treatment effect on the omics data. The small sample size also limits the generalizability of the findings. Despite this limitation, we were able to validate our findings with three independent datasets. Future research using a larger sample size would be needed to determine if our findings are generalizable. Further, it would be interesting to validate our findings with an independent dataset that contains all the molecules we have determined associations with COVID-19 and to find a dataset to validate our COVID-19 status scores from proteins. Often having all views of data for patients is expensive or not possible. The scores we developed were computed for each set of omics data individually, though the molecules were selected through integrative analysis methods. We note that BIPNet and SIDA can still be used for variable selection if there is one view of data and clinical covariates. In such cases, model training can still be performed and scores can be computed for that view. Further, if multi-omics data are available during training but only one type of omics data are available for testing, scores for testing data could still be computed based on variables selected at the training stage which utilized multi-omics data, as we have demonstrated in our validation with independent datasets. The methods we have used handle multi-omic data from the same patients. In a situation where data are obtained from different studies or cohorts, alternative integrative analysis methods would have to be used. When the data come from different studies, the objective of the analysis becomes different than what we explore in this manuscript.

## Conclusions

From the integrative analysis performed on these multi-omics data, we are able to conclude that there is a predictive molecular signature of COVID-19 severity, and a molecular signature that discriminates COVID-19 status. Particularly, we are able to predict disease severity and discriminate disease status using both molecular and pathway scores we developed from proteomic and genomic data. Further we discover through our analysis interesting pathways surrounding immune function and inflammation related to both disease status and severity providing insight to future treatments and therapies for the disease. We are also able to understand the symptoms and consequences of COVID-19 better though discovery of a relationship between non immune function related pathways, some of which are novel discoveries and some of which are corroborated in other research.

## Methods

### Dataset

The data for this multi-omic analysis was collected from April 6, 2020, through May 1 2020 by Overmyer et al., 2020. A total of 128 patients experiencing respiratory issues similar to COVID-19 symptoms were admitted to the Albany Medical Center in Albany, NY, and had blood taken and clinical data collected. Once the blood samples were taken it was determined which patients had the SARS-CoV-2 infection which resulted in 102 patients testing positive for COVID-19, and the remaining 26 patients testing negative. The data from these patients were used to explore the possible correlation of certain biomarkers with COVID-19 status and severity. The blood samples collected were used for multi-omics analyses. RNAseq was performed on leukocytes isolated from the blood samples. From the blood plasma, mass spectrometry (MS) technology was used to identify and quantify proteins, lipids, and metabolites. The data were filtered in two layers described in more detail in the Filtering section. Any molecules which were not significant in either disease status or severity at an alpha of 0.1 were removed from the sample. Following this first layer of filtering, low-variance molecules were removed. The main goal of our paper is to determine which molecules and molecular pathways are key determinants in COVID-19 severity and status. Two methods were used to measure disease severity in the Overmyer et al., 2020 paper. These methods were the World Health Organization (WHO) 0-8 disease-specific scale where 8 denotes death, as well as a score out of 45 indicating the number of hospital-free days (HFD45). An HFD45 value of 0 indicates the individual was still admitted to the hospital after 45 days, or that the individual died. As mentioned in the Overmyer et al., 2020 paper, the scores give comparable outcomes, however, the HFD45 measurement is favoured as it is more granular and not specific to COVID-19. This makes the measure more applicable to the patients who tested negative. For the main analyses in this paper, only clinical covariates present in all of the samples were used. Specifically, we focus on the Charlson comorbidity index (CCI) score, ICU admission status, age, and sex. The CCI score is a score to assess the comorbidities of a patient based on the number and severity of comorbid conditions. Higher scores indicate more comorbidities or higher severity. Comorbidities have been shown to be strongly related to COVID-19 outcomes, so this is a crucial covariate to include in the models [51]. Age has also been shown to have a significant effect on the disease severity [52] so models are adjusted to incorporate age. The initial dataset contains 18,212 genes, 517 proteins, 111 molecules from metabolomics analysis, and 3,357 lipids. The filtering of these molecules is described in the next section. We also use three independent datasets [6, 15, 53] to validate the proteomic and gene scores for severity and status.

### Filtering

To start the filtration process the omics data were transformed with a log base 2 and normalised for each molecule to have mean 0 and standard deviation of 1. For more information on this process please refer to our previous work [8]. We used the data which passed the initial quality control: 517 proteins, 111 molecules from metabolomics analysis, and 3,357 molecules from lipidomics analysis which were read in from the SQLite database (https://www.sqlite.org/index.html). Patients missing observations were excluded from the analysis resulting in a sample of 99 COVID-19 patients and 24 non-COVID-19 patients. In addition, any covariates which were missing more than 70 % of observations were removed from the sample. Any remaining missing variables were imputed via K-nearest neighbourhoods in the “impute” package in R [54] with K=11. The resulting data were then filtered via a univariate regression at an alpha of 0.1. Any molecule which was not statistically significantly associated with COVID-19 or severity was removed from the data. To determine the significance with COVID-19, logistic regression models were fit using COVID-19 status as the outcome. Linear regression models were fit using HFD45 as a continuous response to determining significance with severity. Each molecule was tested for significance using the likelihood-ratio test adjusting for age and sex. This layer of filtering resulted in 14499 genes, 80 metabolites, 352 proteins, and 2031 lipids. The next layer of filtering consisted of removing low-variation molecules. The threshold for low variation was determined separately for each molecule type by analyzing a histogram of the variances. The remaining molecules to be analyzed consist of 5800 genes, 72 molecules from metabolomics analysis, 264 proteins, and 1015 lipids.

### SIDA

Sparse integrative discriminant analysis (SIDA) [11] is an integrative analysis method for jointly modeling associations between two or more data types and separations between classes in each data type. It combines the advantages of linear discriminant analysis (LDA) [55] for maximizing separation between classes in a data type, and canonical correlation analysis (CCA) [56] for maximizing association between two data types. SIDA maximizes the sum of between-class separations (COVID-19 versus non-COVID-19) and the sum of squared correlations between pairs of molecular data. In addition, the method selects important variables that contribute to the association and separation of classes. The optimization problem can be solved using eigensystems. SIDA is able to incorporate prior knowledge of connectivity of molecules (for example molecule networks) by using the normalized Laplacian of a graph to encourage predictors that are connected and behave similarly to be selected. We have chosen this method due to its ability to perform integrative analysis with variable selection, can incorporate clinical covariates, and allow for the use of prior biological information. This way we are likely to determine key molecules associated with COVID-19 status with consideration for molecule functionality. Tuning parameters for sparsity are chosen for each view of data, thus the level of sparsity can be tuned for each view of data separately which is relevant in a situation where there are varying variable dimensions in each view of data. We fit 61 models using the SIDA function in R (link to github [57]), each with a different subset of 60 samples. The left-out sample was used to estimate the test classification error.

### SIDA Scores

A logistic regression model is fit using the training dataset for each molecule selected from SIDA separately, adjusting for clinical covariates age, sex, CCI, and ICU status with COVID-19 status as the outcome to get each molecule’s effect size to create a score. Using the coefficients from the regressions we are able to create scores for the test set individuals by taking the linear combinations of selected molecules with coefficients of the molecules as weights. We create a score from each view separately. We are then able to build a regression model with these scores using the testing dataset adjusting for age, sex, and CCI, and using COVID-19 status as the response in order to assess the discriminatory performance of the scores. In addition, a score for the pathways is created. The molecules selected from SIDA with non-zero coefficients in each of the 61 models undergo Ingenuity Pathway Analysis. Scores are created for each of the pathways and used in regression models to assess their significance in disease status.

### BIPnet

BIPnet is a Bayesian integrative method that combines dataset association and clinical outcome prediction problems [10]. A factor analysis approach is used to integrate the data types and reduce the dimension of datasets to shared components with reduced number of features. Latent variables connect the multiple data types and model the correlations within each data type and between data types, thus inducing a dependency of the data types. BIPnet uses ideas from Bayesian sparse group selection to identify active components in each dataset and important features within components using two nested layers of binary latent indicators. This way the method can account for active shared components and individual components for each datatype. There are three possible scenarios regarding the components: Each component is shared across all data types (scenario only accounts for joint variation), none of the components are shared (scenario only accounts for individual variation), or some components are shared capturing joint structure and individual structure of the data. BIPnet is able to incorporate grouping information (for example gene networks) through prior distributions for variable selection indicator variables, enhancing interpretability. Clinical responses are associated with shared components to evaluate predictive performance. One of the many benefits of this method is clinical covariates can be included without enforcing sparsity. Marginal posterior probabilities (MPPs) are used to determine which components or variables are to be included in the model. An MPP is the probability of a parameter being non-zero unconditional on other parameters. To incorporate grouping information, we used ingenuity pathway analysis (IPA) [58] to determine gene and protein networks that were used in the BIPnet models. This method utilises the MCMC algorithm for posterior inference and a collapsed Gibbs sampling to sample the latent variables and the loadings. We use this method with severity (HFD45) as the response variable and include the clinical covariates CCI, age, sex, and ICU status. We chose this method to analyze molecules associated with COVID-19 severity because it is an integrative analysis method which performs variable selection while considering molecular function through networking information. This method is also favourable to our analysis because it can determine associations between omics and clinical outcomes while incorporating clinical covariates, which is a key objective of this study. BIPnet also accounts for the varying variable dimensions in each view of data by incorporating different prior distributions for variable selection in each view. This means that the level of sparsity for each view can be adjusted to reflect the number of molecules in the view. This is relevant for the dataset analyzed, where there are much more genes and proteins used in analysis than metabolomics and lipidomics. For cross-validation 61 models were fit using the BIPnet package in R [59], (link to github [60]) each with a different subset of 60 samples. The left-out sample was used to estimate the test prediction error.

### Bipnet Score

To create a model using the selected molecules we build univariate regression models using the training data set and each of the molecules selected by BIPnet as a predictor in its own model adjusting for age, sex, and CCI. Using the coefficients from the regressions we are able to create scores for the test set individuals by taking the linear combinations of selected molecules with coefficients of the molecules as weights. We create a score from each view separately. We are then able to build a regression model with these scores adjusting for age, sex, and CCI, and using HFD45 as the response. For these models, the testing dataset is used. In addition to scores for each of the views, we create scores for pathways of molecules found to be significant as determined by IPA. To incorporate the pathways into our regression model we construct a score for each group of genes similar to how we did for each molecule type, where the weights for the molecules are generated using regression with the training set, and the scores are calculated on the testing dataset. In order to not be underpowered, we fit separate regression models using each network score as a predictor. These models once again adjusted for age, sex, and CCI, and used HFD45 as the response. In order to create a score from each view of data, a linear combination of the molecules weighted by the coefficients from the regressions is used on the testing data.

### N-Fold cross-validation

N-fold cross-validation is a popular method for training and assessing the performance of a model. We used leave-one-out cross-validation to determine molecules consistently selected by the integrative analysis methods. This added statistical rigor, facilitating downstream analyses of molecules that are more likely to be associated with disease severity and status. Other resampling techniques (e.g. N-fold cross-validation, bootstrap) could have been used for this purpose but we chose to use leave-one-out cross-validation because we wanted to mitigate against potential loss in information due to our small sample sized data. In this manuscript, the dataset is split into a training dataset of 61 patients and a testing dataset of 62 patients. The BIPnet and SIDA models were fit using n-fold cross-validation on the training dataset. Models were fit for each method 61 times, each leaving out one of the training samples, and a cross-validated error is calculated for each model. The cross-validated error is calculated by taking away the true response from the predicted response of the left-out sample using the corresponding model. The mean cross-validated error gives insight into the performance of the method on the dataset.

## Supplementary Information

**Additional file 1.**

## Data Availability

The metabolomic, proteomic, lipidomic, and clinical datasets analyzed for this study can be found in the MassIVE database: https://doi.org/10.25345/C5F74G. The RNAseq datasets analyzed for this study can be found in GEO: https://www.ncbi.nlm.nih.gov/geo/query/acc.cgi?acc=GSE157103.
