# Supplementary material for: Integrative multi-omics approach for identifying molecular signatures and pathways and deriving and validating molecular scores for COVID-19 severity and status
Source: BMC Genomics. 2023 Jun 12;24:319. doi: 10.1186/s12864-023-09410-5 (PMC10259816; doi:10.1186/s12864-023-09410-5)
Supplement: Supplementary file 1 — Additional file 1. [file 12864_2023_9410_MOESM1_ESM.pdf]

Supplementary Material for " Integrative multi-omics approach for identifying molecular signatures and pathways and deriving and validating molecular scores for COVID-19 severity and status"

May 10, 2023

Supplementary Table 1: Results from IPA on the genes selected with BIPnet. The pathway key is provided which will be used in later tables, as well as the pathway name and molecules which are in the pathway. The p-values are calculated using the right-tailed Fisher's Exact Test. Results are sorted according to significance.

Key	Canonical Pathway	P-value	Molecules
P1	Th1 Pathway	≤0.0001	CD247, CD28, CD3D, CD3E, CD3G, CD4, CD40LG, CD8A, HLA-DPA1, HLA-DQA1, ICOS, IFNG, IL12RB2, KLRD1, NFATC2, PIK3C2A
P2	Th1 and Th2 Activation Pathway	≤0.0001	CD247, CD28, CD3D, CD3E, CD3G, CD4, CD40LG, CD8A, HLA-DPA1, HLA-DQA1, ICOS, IFNG, IL12RB2, IL2RB, KLRD1, NFATC2, PIK3C2A, TGFBR3
P3	Th2 Pathway	≤0.0001	CD247, CD28, CD3D, CD3E, CD3G, CD4, HLA-DPA1, HLA-DQA1, ICOS, IFNG, IL12RB2, IL2RB, NFATC2, PIK3C2A, TGFBR3
P4	ICOS-ICOSL Signaling in T Helper Cells	≤0.0001	CAMK4, CD247, CD28, CD3D, CD3E, CD3G, CD4, CD40LG, HLA-DPA1, HLA-DQA1, ICOS, IL2RB, ITK, ITPR3, LCK, NFATC2, PIK3C2A, PLCG1, PLEKHA1, TRAT1, ZAP70
P5	T Cell Receptor Signaling	≤0.0001	CAMK4, CARD11, CD247, CD28, CD3D, CD3E, CD3G, CD4, CD8A, DUSP5, HLA-DPA1, HLA-DQA1, ICOS, IFNG, ITK, LCK, LEF1, NFATC2, PIK3C2A, PLCG1, RASGRP1, ZAP70
P6	PD-1, PD-L1 cancer immunotherapy pathway	≤0.0001	CD247, CD28, HLA-DPA1, HLA-DQA1, IFNG, IL2RB, LCK, PIK3C2A, PLCG1, RASGRP1, TGFB3, ZAP70
P7	FAK Signaling	≤0.0001	ADGRA2, ADGRA3, ADGRL1, CAPN5, CCR6, CCR7, CD247, CD3D, CD3E, CD3G, CELSR1, CELSR2, CX3CR1, CYSLTR2, EGF, ERBB2, ETS1, GPR15, GPR174, GPR18, GPRC5B, IL12RB2, IL2RB, IL7R, KLF8, LCK, LEF1, P2RY10, PIK3C2A, PLCG1, PTGDR, TGFB3, TGFBR32
P8	Primary Immunodeficiency Signaling	≤0.0001	CD3D, CD3E, CD4, CD40LG, CD8A, ICOS, IL7R, LCK, ZAP70
P9	CD28 Signaling in T Helper Cells	≤0.0001	CAMK4, CARD11, CD247, CD28, CD3D, CD3E, CD3G, CD4, HLA-DPA1, HLA-DQA1, ITK, ITPR3, LCK, NFATC2, PIK3C2A, PLCG1, ZAP70

P10	G-Protein Coupled Receptor Signaling	≤0.0001	ADCY9, ADGRA2, ADGRA3, ADGRL1, AMOT, CAMK4, CCR6, CCR7, CELSR1, CELSR2, CX3CR1, CYSLTR2, DUSP4, GPR15, GPR174, GPR18, GPRC5B, NFATC2, P2RY10, PDE9A, PIK3C2A, PLCG1, PTGDR, RASGRP1, SYNGAP1
P11	CREB Signaling in Neurons	≤0.0001	ADCY9, ADGRA2, ADGRA3, ADGRL1, CAMK4, CCR6, CCR7, CELSR1, CELSR2, CX3CR1, CYSLTR2, EGF, GPR15, GPR174, GPR18, GPRC5B, ITPR3, P2RY10, PIK3C2A, PLCG1, PTGDR, TGFBR3
P12	Crosstalk between Dendritic Cells and Natural Killer Cells	≤0.0001	CCR7, CD28, CD40LG, IFNG, IL2RB, KLRD1, TLR3, TLR7
P13	Phagosome Formation	≤0.0001	ADGRA2, ADGRA3, ADGRL1, CCR6, CCR7, CELSR1, CELSR2, CX3CR1, CYSLTR2, GPR15, GPR174, GPR18, GPRC5B, ITPR3, LCK, P2RY10, PIK3C2A, PLCG1, PTGDR, RASGRP1, TLR3, TLR7, WASF1
P14	Non-Small Cell Lung Cancer Signaling	≤0.0001	E2F5, EGF, ERBB2, FHIT, ITPR3, PIK3C2A, PLCG1, RBL2
P15	Calcium-induced T Lymphocyte Apoptosis	≤0.0001	CAMK4, CD247, CD3D, CD3E, CD3G, CD4, HLA-DPA1, HLA-DQA1, ITPR3, LCK, NFATC2, PLCG1, ZAP70
P16	Role of NFAT in Regulation of the Immune Response	≤0.0001	CAMK4, CD247, CD28, CD3D, CD3E, CD3G, CD4, HLA-DPA1, HLA-DQA1, ITK, ITPR3, LCK, NFATC2, PIK3C2A, PLCG1, ZAP70
P17	Natural Killer Cell Signaling	≤0.0001	CD247, IFNG, IL12RB2, IL2RB, KLRD1, LCK, NFATC2, PIK3C2A, PLCG1, SH2D1A, ZAP70
P18	Protein Kinase A Signaling	≤ 0.0001	ADCY9, AKAP12, CAMK4, CDC14B, DUSP4, DUSP5, ITPR3, LEF1, NFATC2, PDE9A, PLCG1, PTPN13, PTPN4, PTPRK, PTPRM, TGFB3
P19	PKCθ Signaling in T Lymphocytes	≤ 0.0001	CARD11, CD247, CD28, CD3D, CD3E, CD3G, CD4, HLA-DPA1, HLA-DQA1, ITPR3, LCK, NFATC2, PIK3C2A, PLCG1, ZAP70
P20	Breast Cancer Regulation by Stathmin1	≤ 0.0001	ADGRA2, ADGRA3, ADGRL1, CAMK4, CCR6, CCR7, CELSR1, CELSR2, CX3CR1, CYSLTR2, E2F5, EGF, GPR15, GPR174, GPR18, GPRC5B, P2RY10, PIK3C2A, PTGDR, TUBB1

Supplementary Figure 1: Effect sizes determined from univariate regressions of genes selected in BIPnet and used in the score. Colour indicates level of significance.

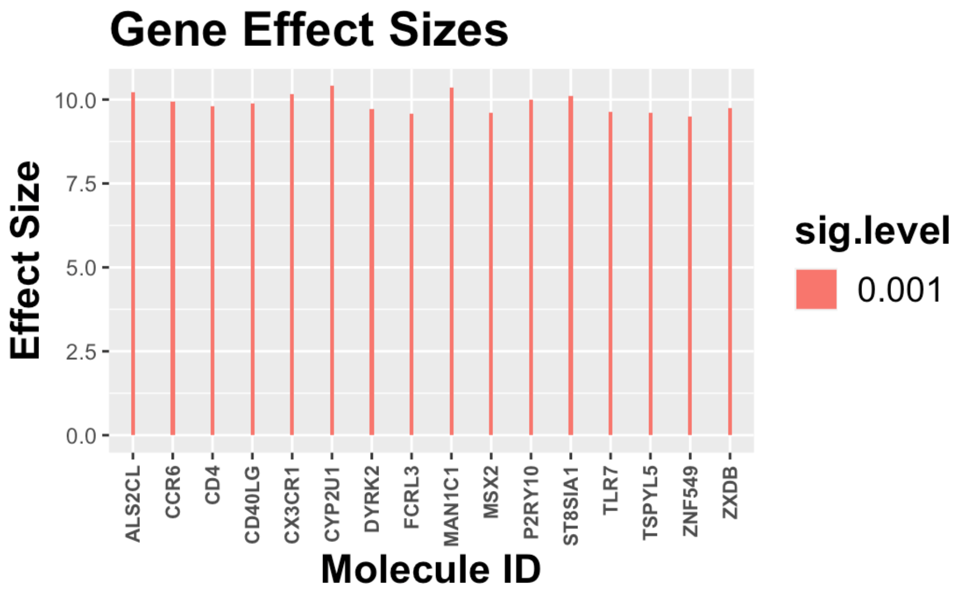

Supplementary Figure 2: Effect sizes determined from univariate regressions of metabolomics molecules selected in BIPnet and used in the score. Colour indicates level of significance.

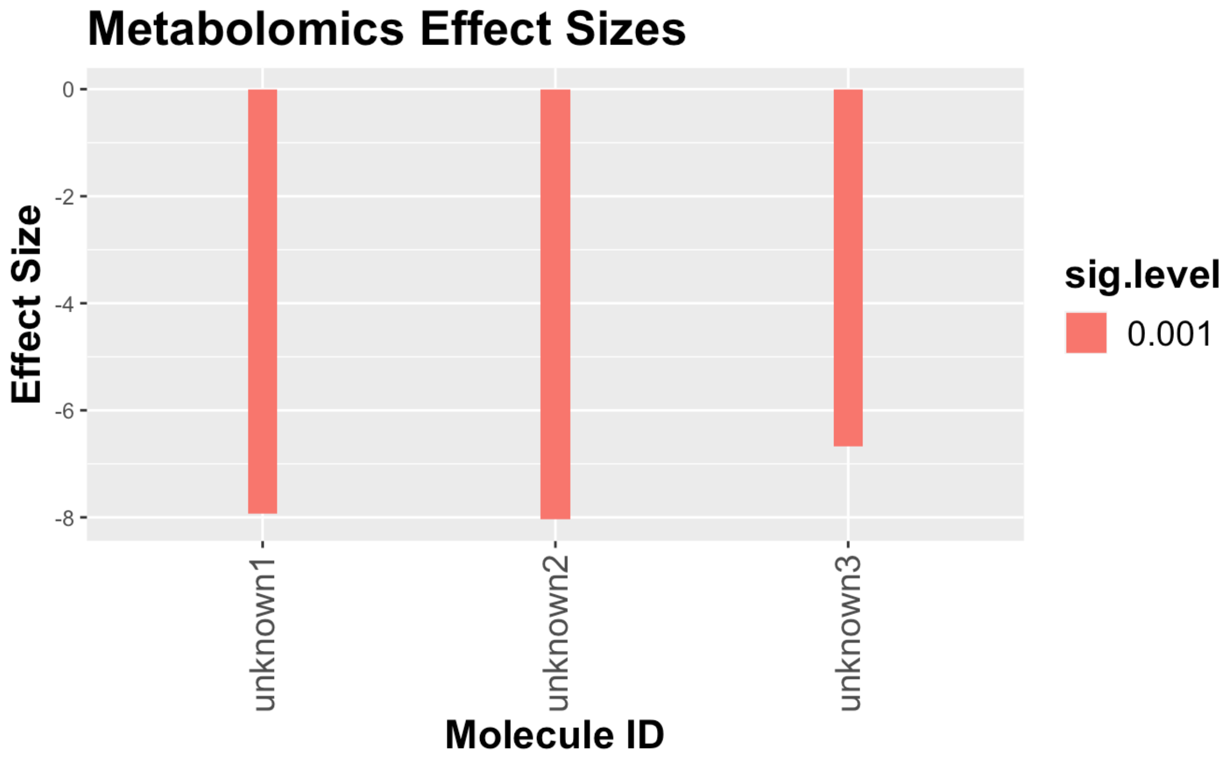

Supplementary Figure 3: Effect sizes determined from univariate regressions of lipidomics molecules selected in BIPnet. Colour indicates level of significance.

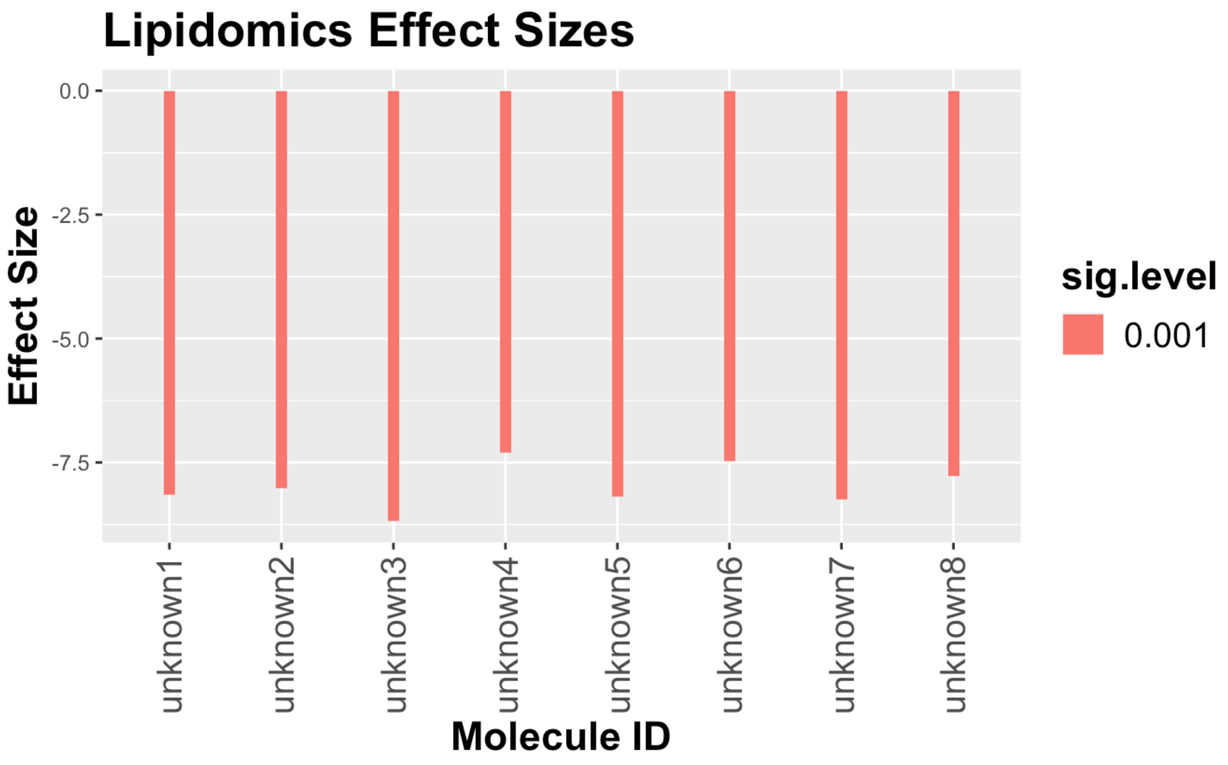

Supplementary Figure 4: Effect sizes determined from univariate regressions of proteins selected in BIPnet. Colour indicates level of significance.

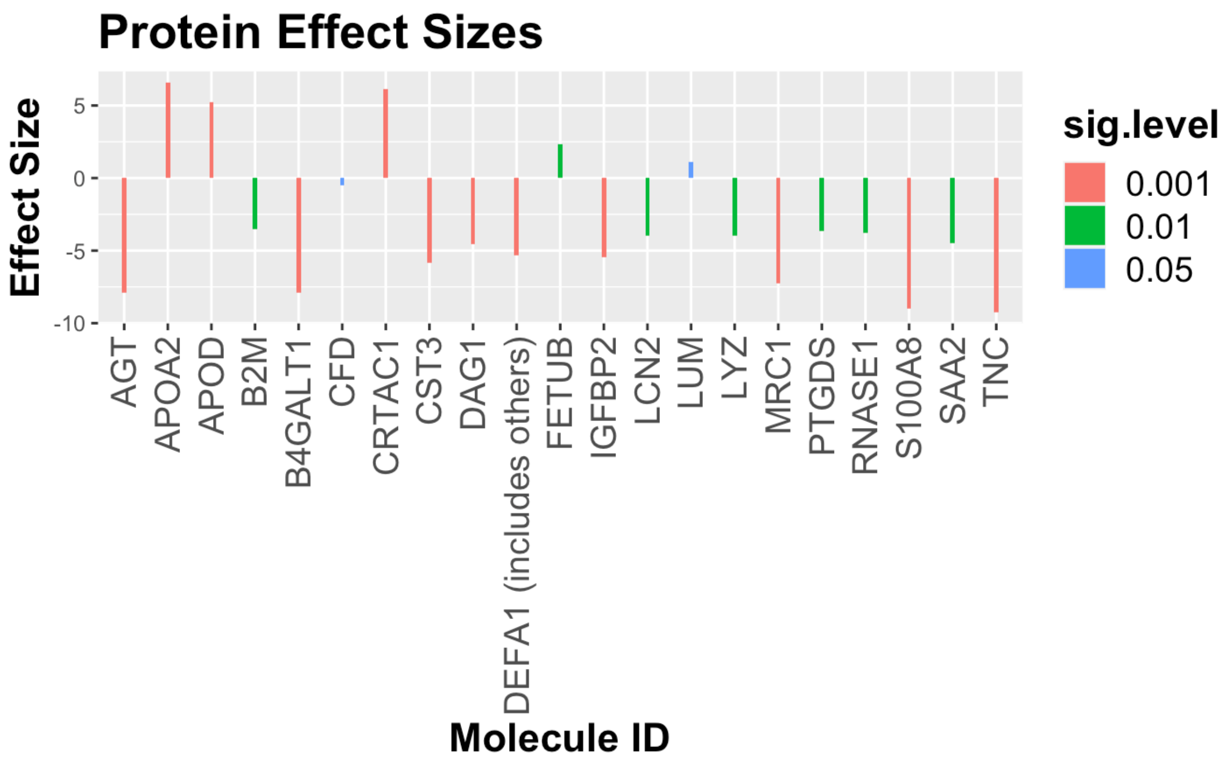

Supplementary Table 2: Results from HFD-45 regressions with gene pathway scores and clinical covariates. Includes MSE, adjusted R squared, the coefficient of the score as well as its p-value, and 95 percent confidence interval. Benjamini Hochberg adjusted p-values were very close to the unadjsuted p-values.

Key(s)	Adjusted R-square	MSE	Coefficient	P-value
P1	0.30	199.97	0.08	$\leq$ 0.0001
P2	0.29	200.83	0.07	$\leq$ 0.0001
P3	0.30	199.34	0.08	$\leq$ 0.0001
P4	0.30	198.22	0.06	$\leq$ 0.0001
P5	0.30	198.06	0.06	$\leq$ 0.0001
P6	0.31	195.41	0.11	$\leq$ 0.0001
P7	0.29	201.02	0.04	$\leq$ 0.0001
P8	0.33	190.18	0.14	$\leq$ 0.0001
P9	0.32	192.71	0.07	$\leq$ 0.0001
P10	0.26	209.52	0.05	$\leq$ 0.0001
P11	0.27	208.14	0.06	$\leq$ 0.0001
P12	0.23	219.51	0.14	0.0003
P13	0.26	210.18	0.06	$\leq$ 0.0001
P14	0.26	209.37	0.24	$\leq$ 0.0001
P15	0.34	186.3	0.09	$\leq$ 0.0001
P16	0.32	191.65	0.08	$\leq$ 0.0001
P17	0.26	209.00	0.11	$\leq$ 0.0001
P18	0.24	216.05	0.08	0.0002
P19	0.33	190.08	0.08	$\leq$ 0.0001
P20	0.26	210.12	0.07	$\leq$ 0.0001

Supplementary Table 3: Results from IPA on the 21 proteins selected with BIPnet. The pathway key is provided which will be used in later tables, as well as the pathway name and molecules which are in the pathway. The p-values are calculated using the right-tailed Fisher's Exact Test. Results are sorted according to significance.

Key	Canonical Pathway	P-value	Molecules
PR1	LXR/RXR Activation	$\leq$0.0001	AGT, APOA2, APOD, LYZ, S100A8, SAA2
PR2	FXR/RXR Activation	$\leq$0.0001	AGT, APOA2, APOD, FETUB, SAA2
PR3	Atherosclerosis Signaling	$\leq$0.0001	APOA2, APOD, LYZ, S100A8
PR4	IL-12 Signaling and Production in Macrophages	$\leq$0.0001	APOA2, APOD, LYZ, S100A8
PR5	Production of Nitric Oxide and Reactive Oxygen Species in Macrophages	$\leq$0.0001	APOA2, APOD, LYZ, S100A8
PR6	Clathrin-mediated Endocytosis Signaling	$\leq$0.0001	APOA2, APOD, LYZ,S100A8

PR7	Airway Pathology in Chronic Obstructive Pulmonary Disease	0.0002	APOD, LCN2, PTGDS
PR8	Acute Phase Response Signaling	0.0006	AGT, APOA2, SAA2
PR9	Maturity Onset Diabetes of Young (MODY) Signaling	0.0023	APOA2, APOD
PR10	Neuroprotective Role of THOP1 in Alzheimer's Disease	0.0048	AGT, CFD

Supplementary Table 4: Results from HFD-45 regressions with protein pathway scores and clinical covariates. Includes MSE, adjusted R squared, the coefficient of the score as well as its p-value.

Key(s)	Adjusted R-square	MSE	Coefficient	P-value	Benjamini Hochberg Adjusted P-values
PR1	0.06	266.63	-0.18	0.1396	0.1629
PR2	0.24	216.82	0.47	0.0002	0.0007
PR3, PR4, PR5, PR6	0.09	259.27	0.32	0.0523	0.0812
PR7	0.08	260.06	0.31	0.0580	0.0812
PR8	0.04	272.43	-0.22	0.3251	0.3251
PR9	0.57	123.21	0.96	≤ 0.0001	≤ 0.0001
PR10	0.12	250.95	-0.45	0.0179	0.0418

Supplementary Table 5: Molecules selected from BIPnet used to create the scores for HFD-45 regression models.

Dataset	Molecules
Protein	CRTAC1, APOD, CFD, AGT, CST3, S100A8, RNASE1, SAA2, B4GALT1, IGFBP2, MRC1, TNC, PTGDS, LUM, DEFA1 , LYZ, B2M, DAG1, FETUB, APOA2, LCN2
Gene	ALS2CL, CCR6, CD4, CD40LG, CX3CR1, CYP2U1, DYRK2, FCRL3, MAN1C1, MSX2, P2RY10, ST8SIA1, TLR7, TSPYL5, ZNF549, ZXDB
Lipid	Unknown, Unknown, Unknown, Unknown, Unknown, Unknown, Unknown, Unknown
Metabolite	Unknown, Unknown, Unknown

Supplementary Table 6: Results from individual HFD-45 regressions with the four view scores and clinical covariates. Includes MSE, adjusted R squared, the coefficient of the score as well as its p-value, and 95 percent confidence interval. Benjamini Hochberg adjusted p-values were very close to the unadjsusted p-values.

Predictor	Coefficient	p-value	95 CI	Adjusted R-square	MSE
Clinical	-	-	-	0.04	277.13
Protein Score + Clinical	0.15	≤ 0.0001	(0.10, 0.20)	**0.40**	169.01
Gene Score + Clinical	0.06	≤ 0.0001	(0.03, 0.09)	**0.34**	200.86
Lipid Score + Clinical	0.13	0.0009	(0.06, 0.21)	0.20	228.41
Metabolite Score + Clinical	0.27	0.0163	(0.05, 0.49)	0.12	250.24
Protein Score	0.16	≤ 0.0001	(0.11, 0.21)	0.41	176.16
Gene Score	0.06	≤ 0.0001	(0.04 0.09)	0.28	213.81
Lipid Score	0.14	0.0004	(0.07, 0.22)	0.18	246.20
Metabolite Score	0.32	0.0021	(0.12, 0.52)	0.13	258.92

Supplementary Table 7: Results from HFD-45 full regression model with all four view scores adjusting for clinical covariates. Includes the coefficient of each score as well as its p-value, and 95 percent confidence interval.

Predictor	Coefficient	p-value	95 CI
Protein Score	0.17	0.0005	(0.08, 0.26)
Gene Score	0.02	0.1602	(-0.01, 0.06)
Lipid Score	0.01	0.8084	(-0.09, 0.12)
Metabolite	-0.29	0.0451	(-0.57, -0.01)

Supplementary Figure 5: Plot of coefficients for genes selected by SIDA determined by logistic regressions with COVID-19 status as the outcome. Genes used to create score: CILP, HLA-G, LCNL1, MMP17, OR52K1, PLPP2, PPP1R17, RNA5S1, VMO1, ILDR1.

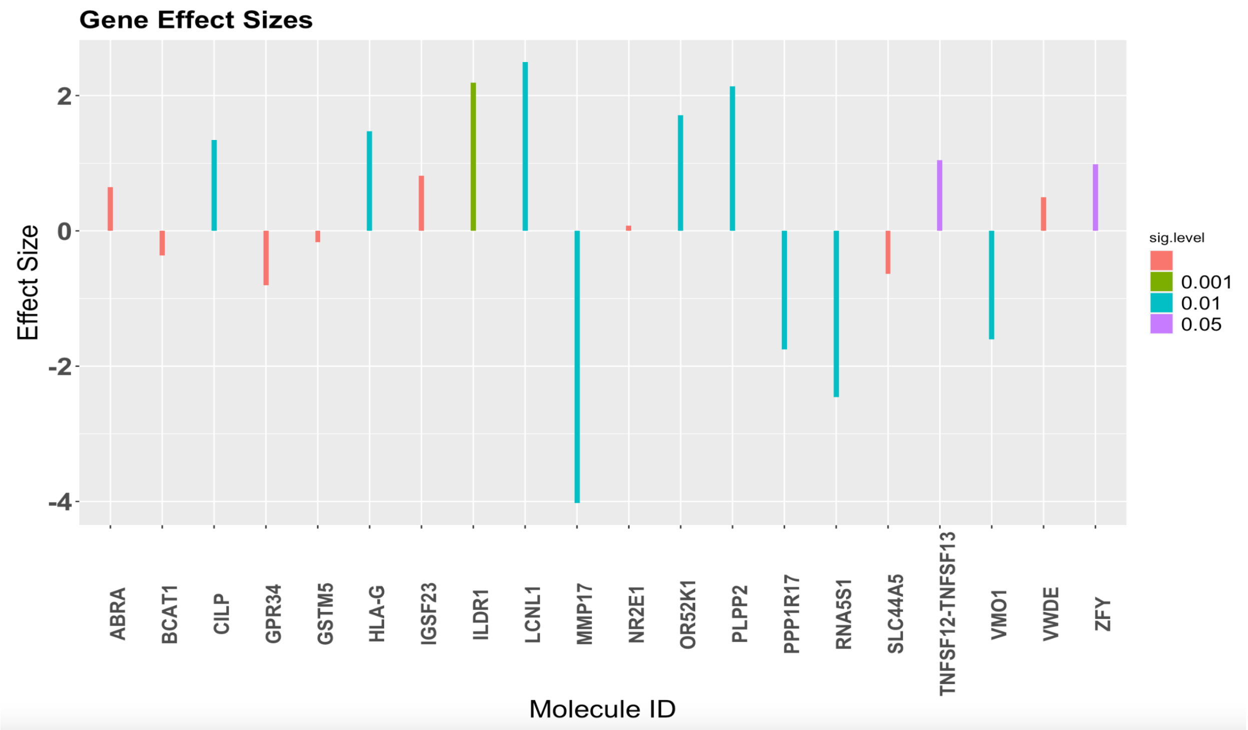

Supplementary Figure 6: Plot of coefficients for metabolomics selected by SIDA determined by logistic regressions with COVID-19 status as the outcome.

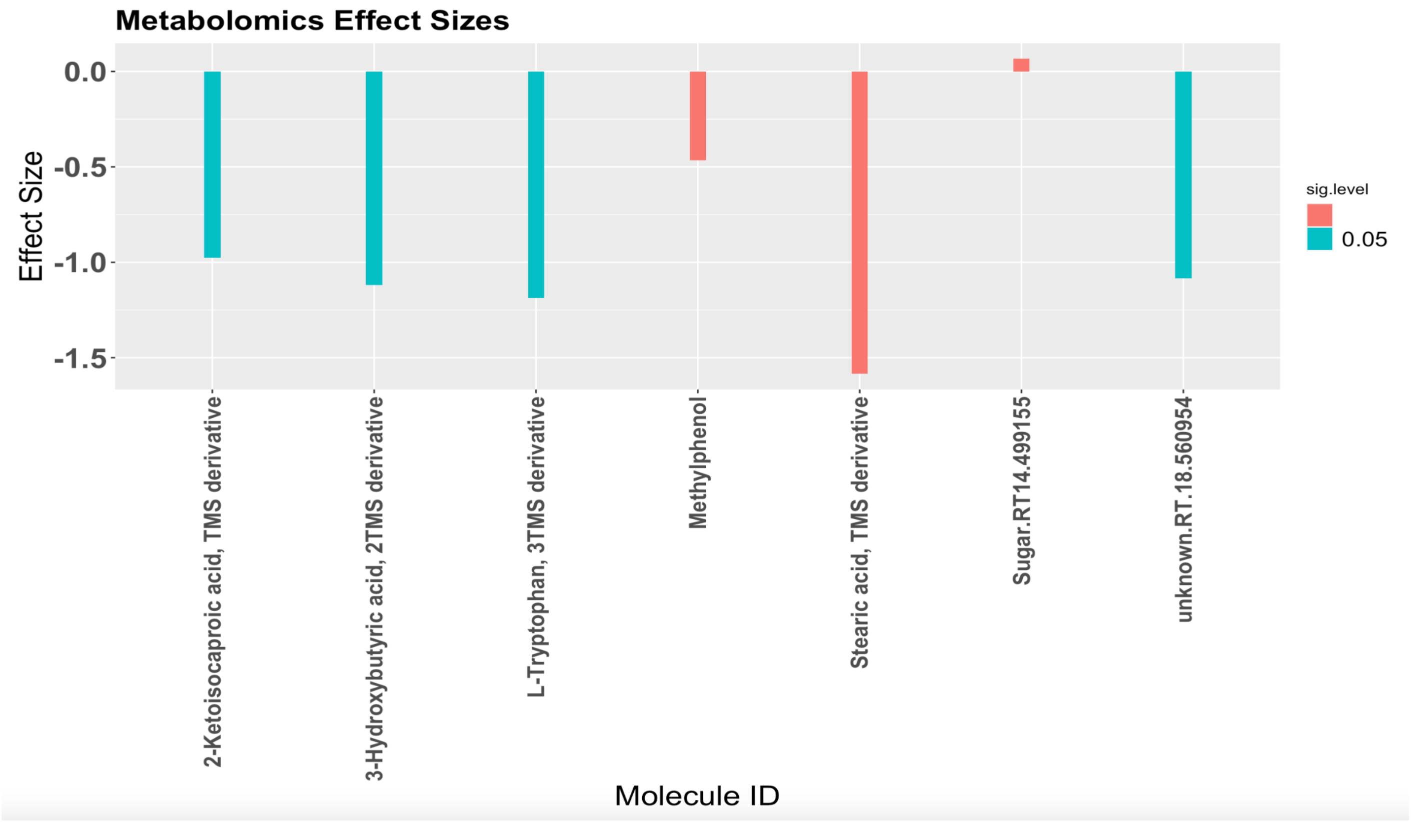

Supplementary Figure 7: Plot of coefficients for lipidomics selected by SIDA determined by logistic regressions with COVID-19 status as the outcome.

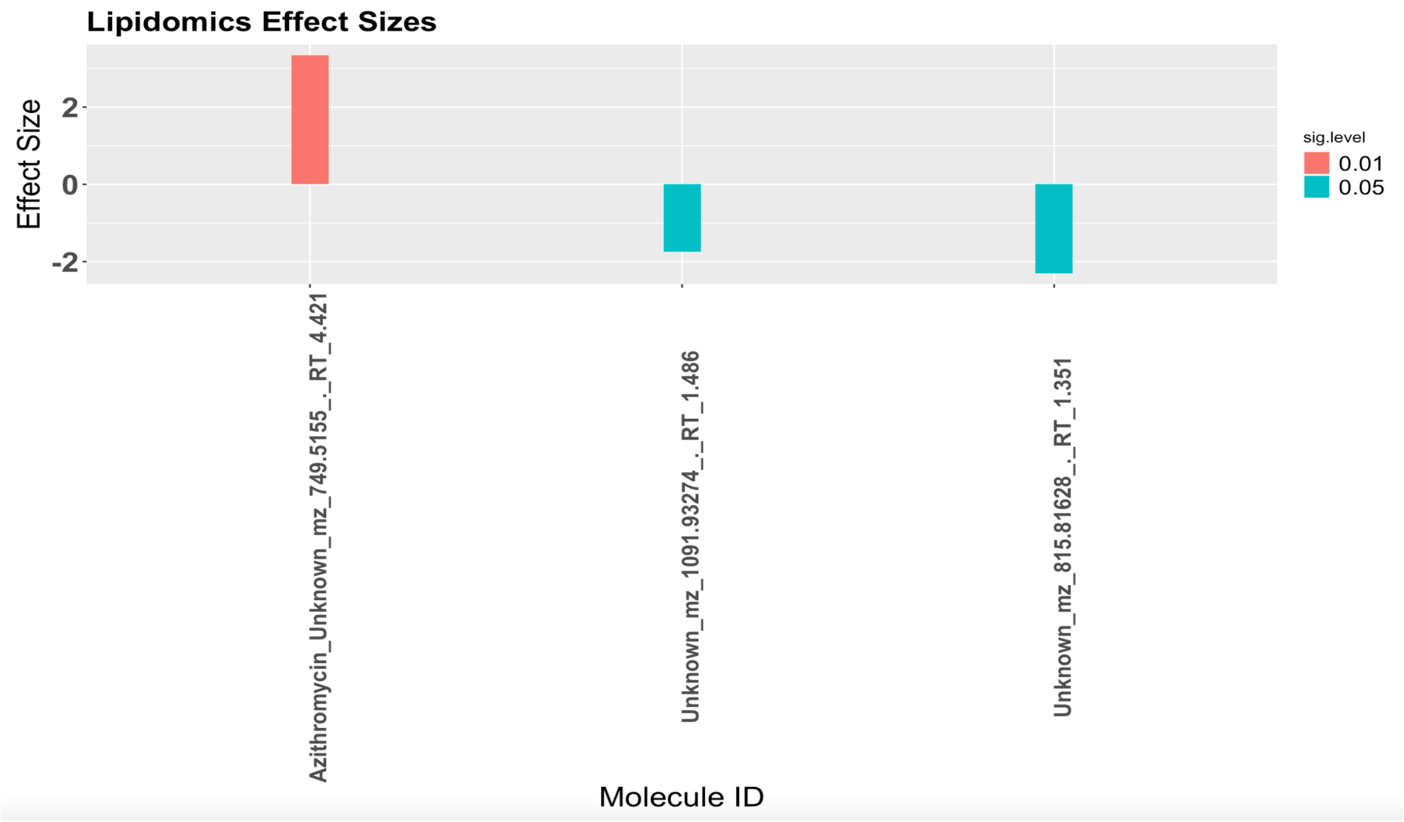

Supplementary Figure 8: Plot of coefficients for proteins selected by SIDA determined by logistic regressions with COVID-19 status as the outcome. Proteins used in creating the score: CHL1, CRTAC1, IGLV3-1, HRG, HSPB1, LCP1, LUM, H3C1, ITIH3, ADIPOQ, APOA2.

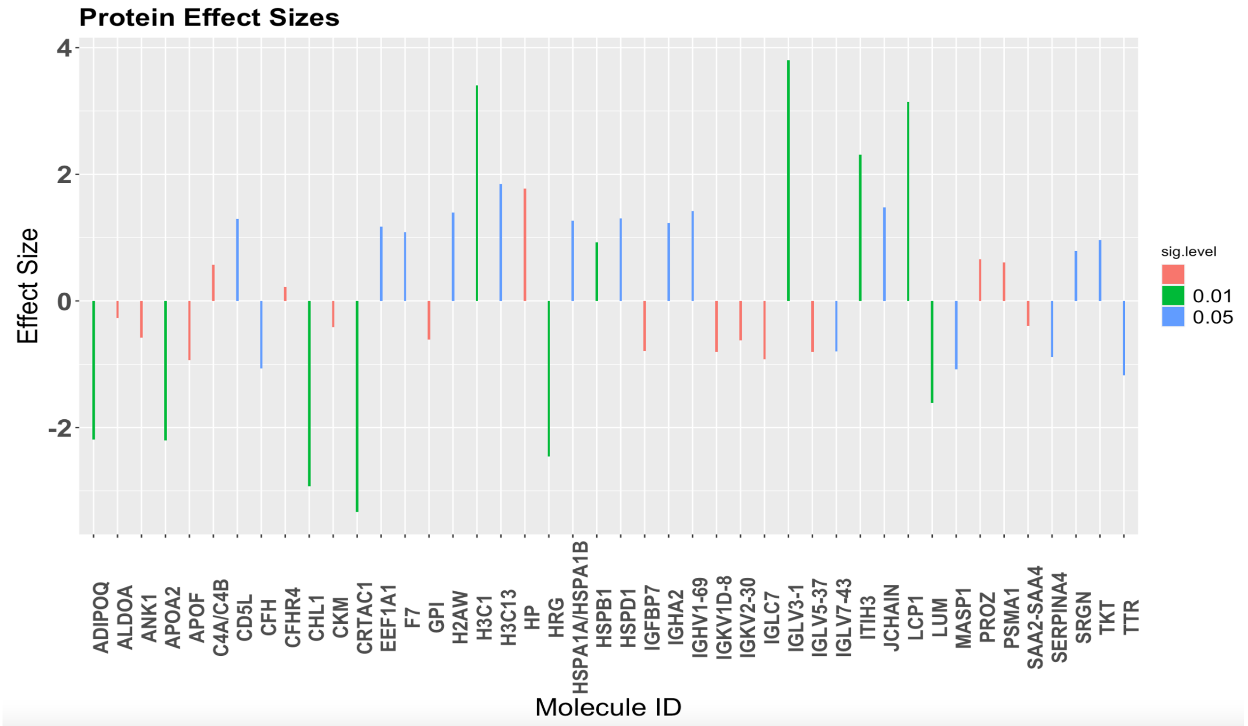

Supplementary Table 8: Results from IPA on the genes selected with SIDA. The pathway key is provided which will be used in later tables, as well as the pathway name and molecules which are in the pathway. The p-values are calculated using the right-tailed Fisher's Exact Test.

Key	Canonical Pathway	P-value	Molecules
P21	Tumor Microenvironment Pathway	0.0107	HLA-G, MMP17

Supplementary Table 9: Results from COVID-19 status logistic regression with gene pathway scores and clinical covariates. Includes AUC, the coefficient of the score as well as its p-value.

Key(s)	AUC	Coefficient	P-value
P21	0.65	0.15	0.1572

Supplementary Table 10: Results from IPA on the proteins selected with SIDA. The pathway key is provided which will be used in later tables, as well as the pathway name and molecules which are in the pathway. The p-values are calculated using the right-tailed Fisher's Exact Test.

Key	Canonical Pathway	P-value	Molecules
PR11	IL-15 Signaling	$\leq$0.0001	IGHA2, IGHV1-69, IGKV1D-8, IGKV2-30, IGLC7, IGLV3-1, IGLV5-37, IGLV7-43
PR12	B Cell Receptor Signaling	$\leq$0.0001	IGHA2, IGHV1-69, IGKV1D-8, IGKV2-30, IGLC7, IGLV3-1, IGLV5-37, IGLV7-43

PR13	Acute Phase Response Signaling	≤0.0001	APOA2, HP, HRG, ITIH3, SAA2-SAA4, TTR
PR14	Systemic Lupus Erythematosus In B Cell Signaling Pathway	≤0.0001	IGHA2, IGHV1-69, IGKV1D-8, IGKV2-30, IGLC7, IGLV3-1, IGLV5-37, IGLV7-43
PR15	Communication between Innate and Adaptive Immune Cells	≤0.0001	IGHA2, IGHV1-69, IGKV1D-8, IGKV2-30, IGLC7, IGLV3-1, IGLV5-37, IGLV7-43
PR16	Maturity Onset Diabetes of Young (MODY) Signaling	0.0005	ADIPOQ, APOA2, APOF
PR17	Glycolysis I	0.0010	ALDOA, GPI
PR18	Gluconeogenesis I	0.0011	ALDOA, GPI
PR19	LXR/RXR Activation	0.0018	APOA2, APOF, TTR
PR20	FXR/RXR Activation	0.0019	APOA2, APOF, TTR
PR21	Complement System	0.0023	CFH, MASP1
PR22	Protein Ubiquitination Pathway	0.0158	HSPB1, HSPD1, PSMA1
PR23	Neuroprotective Role of THOP1 in Alzheimer's Disease	0.0200	F7, MASP1
PR24	Atherosclerosis Signaling	0.0257	APOA2, APOF
PR25	IL-12 Signaling and Production in Macrophages	0.0288	APOA2, APOF
PR26	Aldosterone Signaling in Epithelial Cells	0.0398	HSPB1, HSPD1
PR27	PPARa/RXRa Activation	0.0501	ADIPOQ, APOA2

Supplementary Table 11: Results from COVID-19 status logistic regression with gene pathway scores and clinical covariates. Includes AUC, the coefficient of the score as well as its p-value.

Key(s)	AUC	Coefficient	P-value	Benjamini-Hochberg Pvalues
PR11, PR12, PR14, PR15	.59	0.05	0.6584	0.6584
PR13	0.63	-0.04	0.6584	0.6584
PR16	0.86	-1.31	0.0014	0.0098
PR17, PR18	0.58	0.18	0.5782	0.6584
PR19, PR20	0.70	-0.60	0.07497	0.1749
PR21	0.81	2.87	0.0043	0.0164
PR22	0.58	-0.11	0.2895	0.5084
PR23	0.61	0.11	0.2905	0.5084
PR24, PR25	0.85	-1.49	0.0006	0.0084
PR26	0.76	-1.02	0.0131	0.0367
PR27	0.80	-0.87	0.0047	0.0164

Supplementary Figure 9: Violin plot of densities by COVID-19 status of proteins selected by SIDA in the complement system.

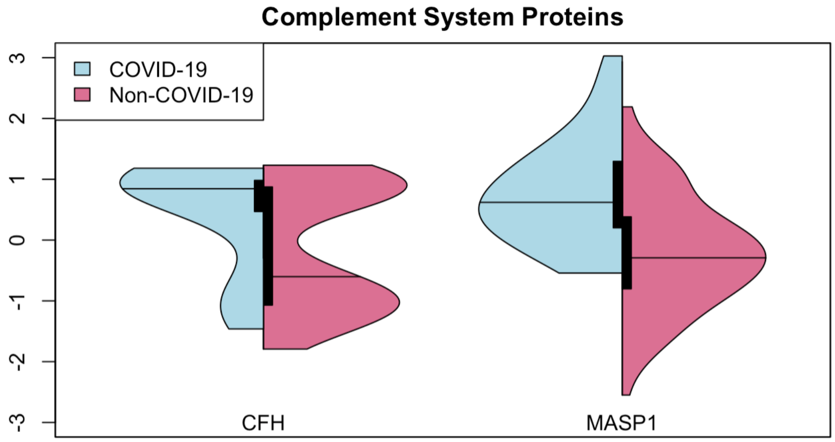

Supplementary Figure 10: Violin plot of densities by COVID-19 status of proteins selected by SIDA in the atherosclerosis signaling pathway and IL-12 signaling and production in macrophages pathway.

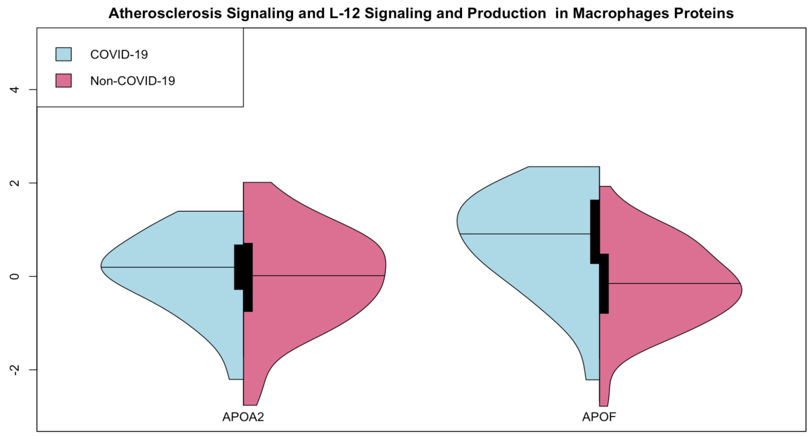

Supplementary Figure 11: Violin plot of densities by COVID-19 status of proteins selected by SIDA in the PARa/RXRa activation pathway.

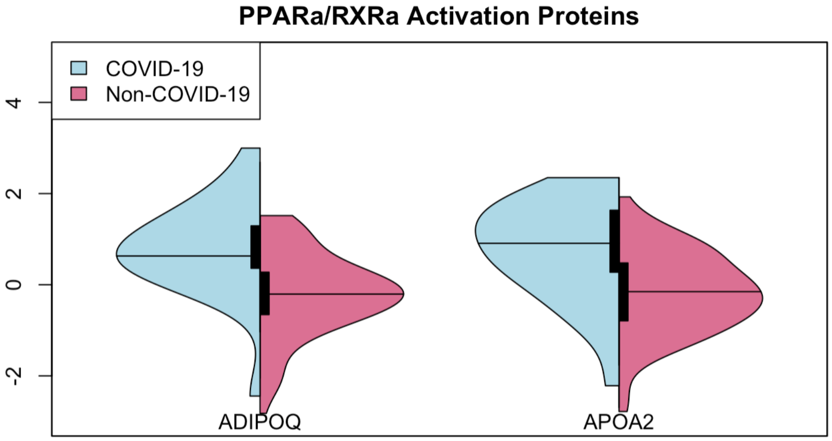

Supplementary Table 12: Molecules used to create the scores for COVID-19 status logistic regression models.

Dataset	Molecules
Protein	CHL1, CRTAC1, IGLV3-1, HRG, HSPB1, LCP1, LUM, H3C1, ITIH3, ADIPOQ, APOA2
Gene	CILP, HLA-G, LCNL1, MMP17, OR52K1, PLPP2, PPP1R17, RNA5S1, VMO1, ILDR1
Lipid	Unknown, Unknown, Azithromycin
Metabolite	Methylphenol, 3-Hydroxybutyric acid 2TMS derivative, 2-Ketoisocaproic acid TMS derivative, Sugar, unknown, L-Tryptophan 3TMS derivative, Stearic acid TMS derivative

Supplementary Figure 12: Violin plot of densities by COVID-19 status of lipids selected by SIDA.

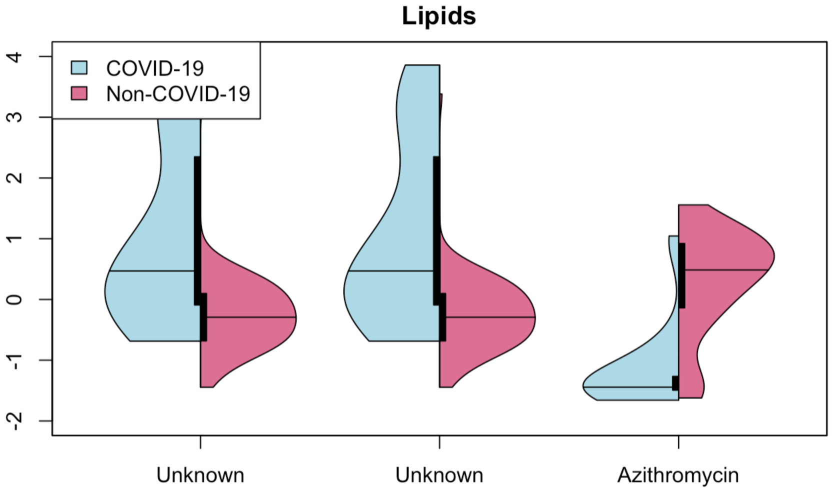

Supplementary Figure 13: Violin plot of densities by COVID-19 status of metabolites selected by SIDA.

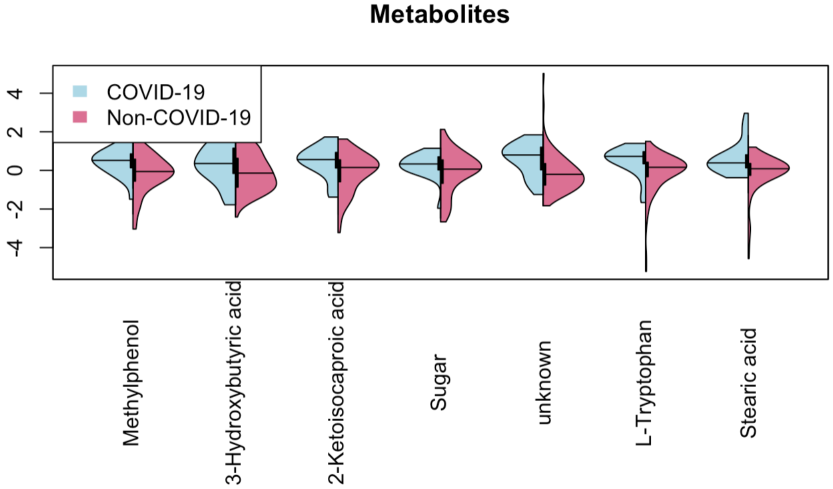

Supplementary Figure 14: Violin plot of densities by COVID-19 status of proteins selected by SIDA.

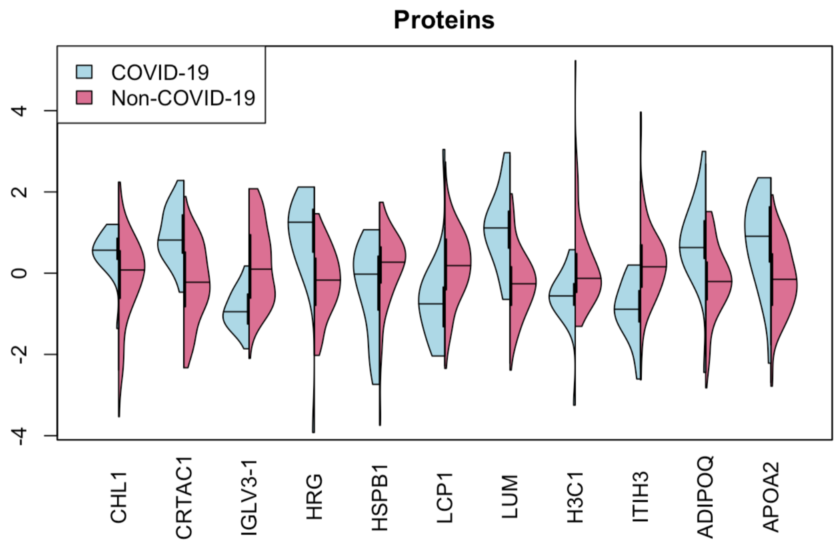

Supplementary Figure 15: Violin plot of densities by COVID-19 status of genes selected by SIDA.

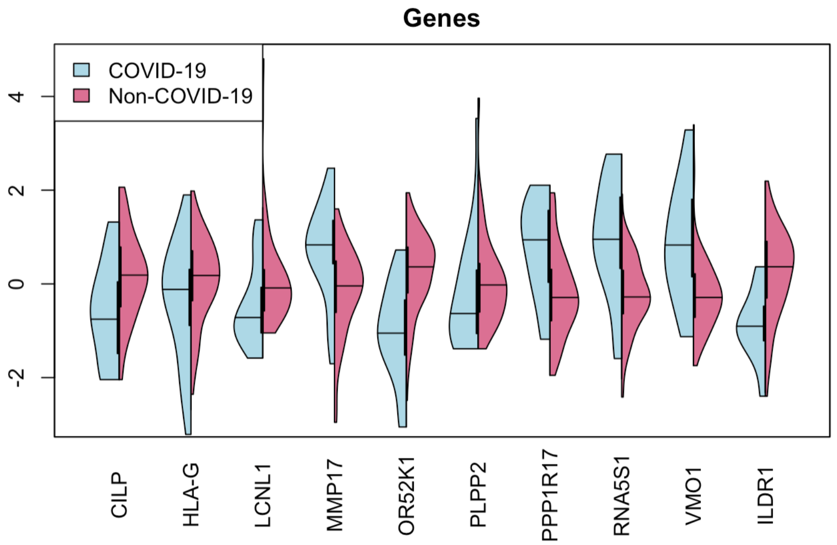

Supplementary Table 13: Results from individual COVID-19 status logistic regressions with the four view scores and clinical covariates. Includes AUC, the coefficient of the score as well as its p-value, and 95 percent confidence interval. Note that none of the clinical covariates were significant at a level of 0.05.

Predictor	Coefficient	p-value	95 CI	AUC
Clinical	-	-	-	0.59
Protein Score	0.14	≤ 0.0001	(0.07, 0.23)	**0.90**
Gene Score	0.15	0.0014	(0.07,0.25)	0.77
Lipid Score	0.29	0.0012	(0.15, 0.51)	**0.90**
Metabolite	0.43	0.0025	(0.18 ,0.74)	0.79
Protein Score + Clinical	0.16	0.0004	(0.08, 0.26)	**0.90**
Gene Score + Clinical	0.17	0.0011	(0.08, 0.28)	0.82
Lipid Score + Clinical	0.53	0.0013	(0.18, 0.63)	**0.93**
Metabolite + Clinical	0.53	0.0030	(0.23, 0.94)	0.82

Other Points of Discussion

We determined that many pathways which are related to immune function are enriched in COVID-19. First, we discuss the Th1 and Th2 pathways. Note that three separate pathways surrounding Th1 and Th2 were found to be significant from the BIPnet selected genes: Th1 pathway, Th1, and Th2 activation pathway, and Th2 pathway. These pathways were examined in other research, where it was found that there is an association between Th1 and Th2 and COVID-19 severity[1]. Th1 and Th2 are molecules that play a role in the body's inflammatory response to disease. The paper cited found that Th2 and Th1 cytokine imbalance is associated with major COVID-19 risk factors. Particularly, this paper concluded that the imbalance was more evident in patients with asthma who died from COVID-19. The scores from all three of these pathways were found to be significant in the HFD-45 regression models.

Another pathway-related immune system function we found to be significant from BIPnet and IPA is the primary immunodeficiency signaling pathway. This association with COVID-19 severity is to be expected, as lots of research points to individuals with immunodeficiency experiencing more severe cases[2]. Other pathways surrounding immune function associated with COVID-19 severity are natural killer cell signaling and cross-talk between dendritic cells and natural killer cells.

Other pathways we determined to be associated with COVID-19 that are related to immune system function

are PPARa/RXRa activation pathway and pathways surrounding B cell processes: systemic lupus erythematosus in B cell signaling, and B cell receptor signaling pathways.

Other pathways we found significant from the proteins selected in BIPnet are g-Protein coupled receptor signaling and the clathrin-mediated endocytosis signaling pathway. We also determined that airway pathology in the chronic obstructive pulmonary disease pathway is associated with COVID-19 severity. Chronic obstructive pulmonary disease (COPD) occurs from an inflammatory immune response to the inhalation of toxic particles and gases. Tobacco, though not the only cause of this, is the primary cause[3]. This pathway being associated with COVID-19 severity could be pointing toward the relationship between smoking and disease severity.

References

[1] Pavel, A.B., Glickman, J.W., Michels, J.R., Kim-Schulze, S., Miller, R.L., Guttman-Yassky, E.: Th2/th1 cytokine imbalance is associated with higher covid-19 risk mortality. Front Genet **12**, 706902 (2021). doi:10.3389/fgene.2021.706902

[2] Liu, B.M., Hill, H.R.: Role of host immune and inflammatory responses in covid-19 cases with underlying primary immunodeficiency: A review. J Interferon Cytokine Res **40**(12), 549–554 (2020). doi:10.1089/jir.2020.0210

[3] Hogg, J.C., Timens, W.: The pathology of chronic obstructive pulmonary disease. Annu Rev Pathol **4**, 435–459 (2009). doi:10.1146/annurev.pathol.4.110807.092145
